# RUNX1 colludes with NOTCH1 to reprogram chromatin in T cell acute lymphoblastic leukemia

**DOI:** 10.1016/j.isci.2023.106795

**Published:** 2023-05-03

**Authors:** Rashedul Islam, Catherine E. Jenkins, Qi Cao, Jasper Wong, Misha Bilenky, Annaïck Carles, Michelle Moksa, Andrew P. Weng, Martin Hirst

**Affiliations:** 1 Bioinformatics Graduate Program, University of British Columbia, Vancouver, BC V5Z 4S6, Canada; 2 Department of Microbiology and Immunology, Michael Smith Laboratories, University of British Columbia, Vancouver, BC V6T 1Z3, Canada; 3 Canada’s Michael Smith Genome Sciences Centre, BC Cancer, Vancouver, BC V5Z 4S6, Canada; 4 Terry Fox Laboratory, BC Cancer, Vancouver, BC V5Z 1L3, Canada; 5 Genome Science and Technology Program, University of British Columbia, Vancouver, BC V6T 2B5, Canada; 6 Department of Pathology and Laboratory Medicine, University of British Columbia, Vancouver, BC V6T 2B5, Canada

**Keywords:** Molecular biology, Cancer systems biology, Cancer

## Abstract

Runt-related transcription factor 1 (RUNX1) is oncogenic in diverse types of leukemia and epithelial cancers where its expression is associated with poor prognosis. Current models suggest that RUNX1 cooperates with other oncogenic factors (e.g., NOTCH1, TAL1) to drive the expression of proto-oncogenes in T cell acute lymphoblastic leukemia (T-ALL) but the molecular mechanisms controlled by RUNX1 and its cooperation with other factors remain unclear. Integrative chromatin and transcriptional analysis following inhibition of RUNX1 and NOTCH1 revealed a surprisingly widespread role of RUNX1 in the establishment of global H3K27ac levels and that RUNX1 is required by NOTCH1 for cooperative transcription activation of key NOTCH1 target genes including *MYC, DTX1, HES4, IL7R,* and *NOTCH3*. Super-enhancers were preferentially sensitive to RUNX1 knockdown and RUNX1-dependent super-enhancers were disrupted following the treatment of a pan-BET inhibitor, I-BET151.

## Highlights

• RUNX1 regulates H3K27ac density genome-wide and within super-enhancers
• RUNX1 cooperates with NOTCH1 to drive an oncogenic gene expression program
• RUNX1 dependent super-enhancers are disrupted by I-BET151 treatment
• RUNX1 mediated chromatin remodeling contributes to the disruption of G1-S checkpoint

## Graphical abstract

**Figure:**
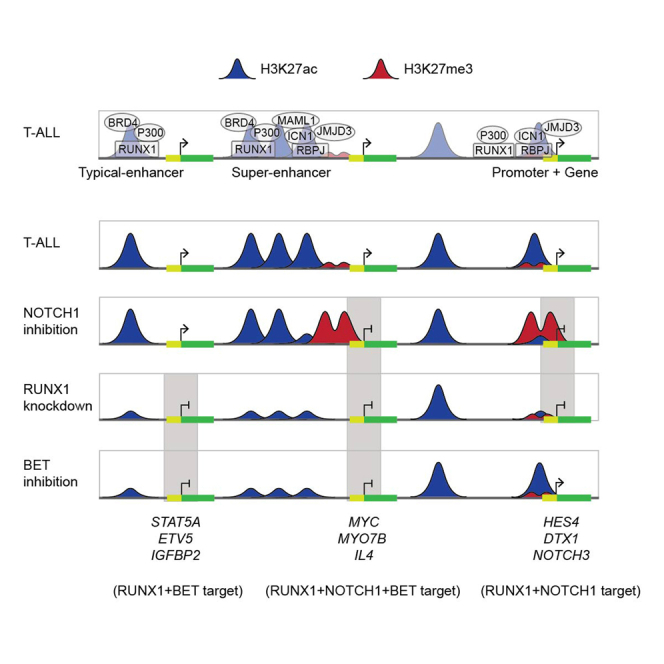

## Introduction

Since the cloning of runt-related transcription factor 1 (*RUNX1*) over 30 years ago significant effort has been directed to understanding its functional role in hematopoietic and epithelial cancers where *RUNX1* translocation, mutation, copy number gain, and over-expression have been described.^1^^,^^2^^,^^3^ The RUNX family of transcription factors cf. DNA binding capacity to the heterodimeric core binding factor (CBF) transcription complex.^4^ Members of the CBF complex are commonly perturbed during leukemogenesis and are frequent targets of translocation in T cell acute lymphoblastic leukemia (T-ALL), myelodysplastic syndromes (MDS), acute myeloid leukemia (AML), and B-cell acute lymphoblastic leukemia (B-ALL)^5^^,^^6^^,^^7^ Missense mutations within the DNA binding domain of RUNX1 are recurrent events in T-ALL (10–15% cases) suggesting a tumor suppressor role.^8^^,^^9^^,^^10^ This interpretation is complicated by the observation that *RUNX1* is overexpressed in T-ALL and required for leukemic cell growth and survival in murine T-ALL models.^11^^,^^12^^,^^13^

T-ALL accounts for 15% of pediatric and 25% of adult acute lymphoblastic leukemia cases.^14^ T-ALL subgroups are characterized by the arrest of thymocytes at different stages of development and each subgroup can be classified by the over-expression or genetic activation of specific transcription factors.^15^^,^^16^ Independent of T-ALL subgroups, more than 55% of T-ALL cases have recurrent gain-of-function mutations in *NOTCH1*^17^^,^^18^ in the background of expressed RUNX1.^10^ Following nuclear translocation, NOTCH1 is thought to co-bind enhancer elements with RUNX1 to drive expression of key NOTCH1 target genes in T-ALL.^11^^,^^19^ Cooperativity between Runx and Notch appears to be conserved in *Drosophila* hemocytes, where Runx is required for Notch responsive enhancers to be competent to respond to Notch.^20^

RUNX1 drives expression of key myeloid differentiation genes (e.g., *PU.1, MPO, GM-CSF*) and its recruitment to DNA induces an active chromatin state including acetylation of histone 3 lysine 27 (H3K27ac) and, in the context of gene promoters, trimethylation of histone lysine 4 (H3K4me3).^21^^,^^22^^,^^23^ RUNX1 itself does not possess enzymatic activity but serves to recruit histone acetyltransferases (CBP/P300) and lysine methyltransferases (MLL) to deposit H3K27ac and H3K4me3 on nearby histones, respectively.^21^^,^^22^ Interaction with CBP/P300 also leads to acetylation of RUNX1 that enhances its DNA binding activity.^24^ RUNX1 recruitment to, and activation of, gene promoters provide a direct mechanism of transcriptional activation. However, two-thirds of the total (20,000–30,000) RUNX1 binding sites are located outside of promoters^19^^,^^25^ and the contribution of RUNX1 to the chromatin landscape more broadly is not known.

Gain-of-function mutation of NOTCH1 is a defining feature of T-ALL^18^ and is thought to act in part through disruption of polycomb repressive complex 2 (PRC2) mediated repression.^26^ The NOTCH1 intracellular domain (ICN1) directly interacts with the H3K27me3 demethylase, JMJD3, and through its recruitment to NOTCH1 binding elements is thought to oppose PRC2 mediated transcriptional repression.^26^^,^^27^ A tumor-suppressive role for PRC2 in T-ALL is reinforced by the observation of frequent loss-of-function mutations in the members of the complex.^27^ But the specific role of NOTCH1 mediated H3K27me3 loss in T-ALL has yet to be established.

To examine the contribution of RUNX1 and NOTCH1 in the establishment of a pathogenic transcriptional signature in T-ALL, we performed an integrative analysis of epigenetic and transcriptional states following RUNX1 knockdown and NOTCH1 inhibition. Analysis of these data revealed that RUNX1 cooperates with NOTCH1 to establish active chromatin states and drive the expression of key T-ALL oncogenes. RUNX1 regulates H3K27ac occupancy genome-wide and is required to maintain a majority of super-enhancers in T-ALL, including its own intronic super-enhancer that drives a feedforward transcriptional loop. Bromodomain and extraterminal (BET) proteins (BRDT, BRD2, BRD3, and BRD4) are the epigenetic reader of H3K27ac modification and are themselves recruited to super-enhancers.^28^ Treatment with the pan-BET protein inhibitor I-BET151 led to a broad reduction in H3K27ac levels, disrupted RUNX1 driven pathogenic super-enhancers, and down-regulated key RUNX1 and NOTCH1 target genes and proliferation of T-ALL cell lines. Our results indicate that RUNX1 plays a surprisingly broad role in the maintenance of H3K27ac density at super-enhancers and suggests that targeted disruption of super-enhancers through I-BET151 treatment may provide therapeutic benefit in RUNX1-driven malignancies.

## Results

**Figure 1:**
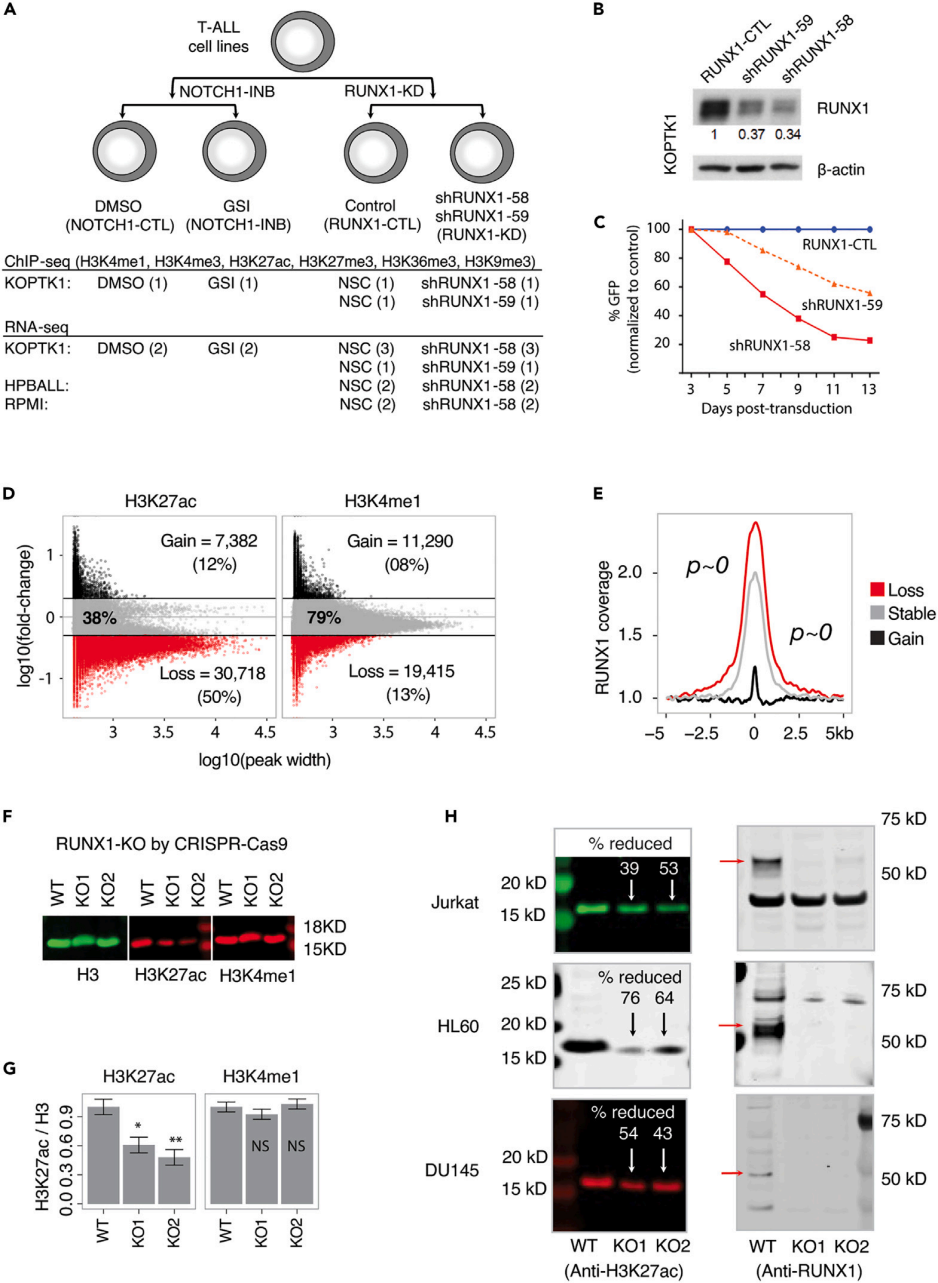
RUNX1-KD is associated with a genome-wide reduction in H3K27ac (A) Experimental strategy of NOTCH1 inhibition (NOTCH1-INB) and *RUNX1* knockdown (RUNX1-KD). NSC means non-silencing control and GSI means gamma secretase inhibitor. The number of replicates is indicated in brackets. (B) Anti-RUNX1 Western blot of protein levels following knockdown by shRUNX1-58 and shRUNX1-59. Numbers below the RUNX1 panel indicate their relative expression level after normalization to β-actin loading control. (C) Growth competition assays showing the fraction of GFP+ cells following transduction with GFP-tagged lentiviruses expressing shRUNX1s and normalized to a scrambled shRNA control (RUNX1-CTL). (D) Normalized read densities of H3K27ac and H3K4me1 peaks following RUNX1-KD (red ≥ 2-fold loss; black ≥2-fold gain; and gray unchanged). Read densities were normalized by sequencing depth. (E) Mean RUNX1 ChIP-seq read densities at gained, lost, and stable H3K27ac regions following RUNX1-KD. RUNX1 ChIP-seq data was derived from CUTLL1 and p values were calculated using KS-test. (F) Western blot of histone H3, H3K27ac, and H3K4me1 levels in control (RUNX1-WT) and RUNX1-KOs (KO1 and KO2) using two CRISPR guides in KOPTK1. (G) Ratio of H3K27ac and H3K4me1 relative to the H3 control was quantified from the protein bands shown in (F). Three independent experiments were performed to calculate p values using unpaired two-tailed t-test where ∗ indicates p value ≤ 0.05 and ∗∗ for p value ≤ 0.01. Data are mean ± s.e.m. of H3K27ac/H3. (H) Western blot analysis of K3K27ac and RUNX1 in control (RUNX1-WT) and RUNX1-KOs (KO1 and KO2) in Jurkat, HL60, and DU145 cell lines. Where shown, percent reduction was calculated by comparing control (RUNX1-WT) to the knockout samples.

To dissect the molecular mechanisms of RUNX1 and its relationship with NOTCH1, we performed histone ChIP-seq targeting six histone modifications (H3K4me1, H3K4me3, H3K9me3, H3K27me3, H3K36me3, and H3K27ac) in a T-ALL cell line (KOPTK1) following short hairpin RNA knockdown of *RUNX1* (RUNX1-KD) or treatment with pharmacological inhibitor (Gamma secretase inhibitor [GSI]) of Notch signaling (NOTCH1-INB) (Figures 1A, S1A–S1C and Table S1). We assessed transcriptome changes by RNA-seq following NOTCH1-INB in KOPTK1, and RUNX1-KD in KOPTK1, HPBALL, and RPMI T-ALL cell lines (Figure 1A). To confirm that our GSI treatment effectively blocked NOTCH1 activity, we examined the expression of known NOTCH1 target genes and confirmed their downregulation (Figure S1D). We examined RUNX1 protein levels following RUNX1-KD using two independent shRNAs (shRUNX1-58 and shRUNX1-59). Western blot analysis confirmed robust reduction in RUNX1 protein levels with shRUNX1-58 showing higher efficiency than shRUNX1-59 (Figure 1B) and thus we selected shRUNX1-58 to characterize the molecular consequences of RUNX1-KD. Consistent with previous reports,^11^^,^^12^ both shRNA constructs reduced KOPTK1 cell proliferation (Figure 1C).

### RUNX1 contributes to H3K27ac occupancy genome-wide

To quantitate histone modification changes following RUNX1-KD, we called enriched regions (peaks) from the ChIP-seq datasets using FindER^29^ and generated a union of peaks from RUNX1-CTL and RUNX1-KD samples. Normalized read densities were used to identify the peaks that show a 2-fold change between RUNX1-CTL and RUNX1-KD. Among the six histone modifications, H3K27ac demonstrated the most significant reduction following RUNX1-KD with 50% of peaks losing H3K27ac density (Figures S1E and 1D). In contrast to the genome-wide loss observed for H3K27ac (marking active enhancers), occupancy of H3K4me1 (primed enhancers) remained largely stable following RUNX1-KD (Figure 1D). RUNX1-dependent H3K27ac peaks were highly enriched with RUNX1 ChIP-seq signal compared to the peaks gained or stable upon RUNX1-KD (Figure 1E). To replicate the genome-wide dependence of H3K27ac levels on RUNX1, we performed H3K27ac ChIP-seq following RUNX1-KD in an independent T-ALL cell line (CUTLL1) using a third shRNA (shRUNX1-90). Despite the variations observed in knockdown efficiencies of shRUNX1 constructs, there were significant overlaps (hypergeometric p value ∼0) of H3K27ac peaks lost using independent shRUNX1 constructs across two T-ALL cell lines (Figures 1D, S2A, and S2B). The shRNA-associated H3K27ac density reduction varied across cell lines to a greater degree than within a single line. However, in all treatments a reduction in H3K27ac density was observed.

To validate the role of RUNX1 in the maintenance of genome-wide H3K27ac levels, we quantified bulk H3K27ac levels from histones extracted following CRISPR-Cas9 mediated disruption of the DNA binding Runt domain of *RUNX1* using two guide RNAs to generate two independent knockout models (RUNX1-KO1 and RUNX1-KO2) (Figures S2C and S2D; see STAR Methods). Sequencing of the gRNA target sites showed ∼90% of RUNX1 knockout efficiency in the bulk population of cells and we observed an expected growth arrest following RUNX1 knockout (RUNX1-KO) (Figures S2D and S2E). Measurement of global H3K27ac levels by western blot confirmed a significant reduction (∼50%) in H3K27ac upon RUNX1-KO whereas H3K4me1 remained unchanged in KOPTK1 as expected (Figures 1F and 1G). To expand on this observation, we performed CRISPR-Cas9 mediated RUNX1-KO in another T-ALL cell line (Jurkat), AML (HL60) and prostate adenocarcinoma (DU145) cell lines, contexts where increased RUNX1 expression is associated with disease progression.^30^^,^^31^^,^^32^ Consistent with the KOPTK1 observations, RUNX1-KO led to ∼50% reduction of H3K27ac levels in all three cell lines (Figures 1H and S3A–S3D) suggesting that RUNX1 associated loss of genome-wide H3K27ac density may be generalizable across cancer types.

### RUNX1 establishes oncogenic super-enhancers

**Figure 2:**
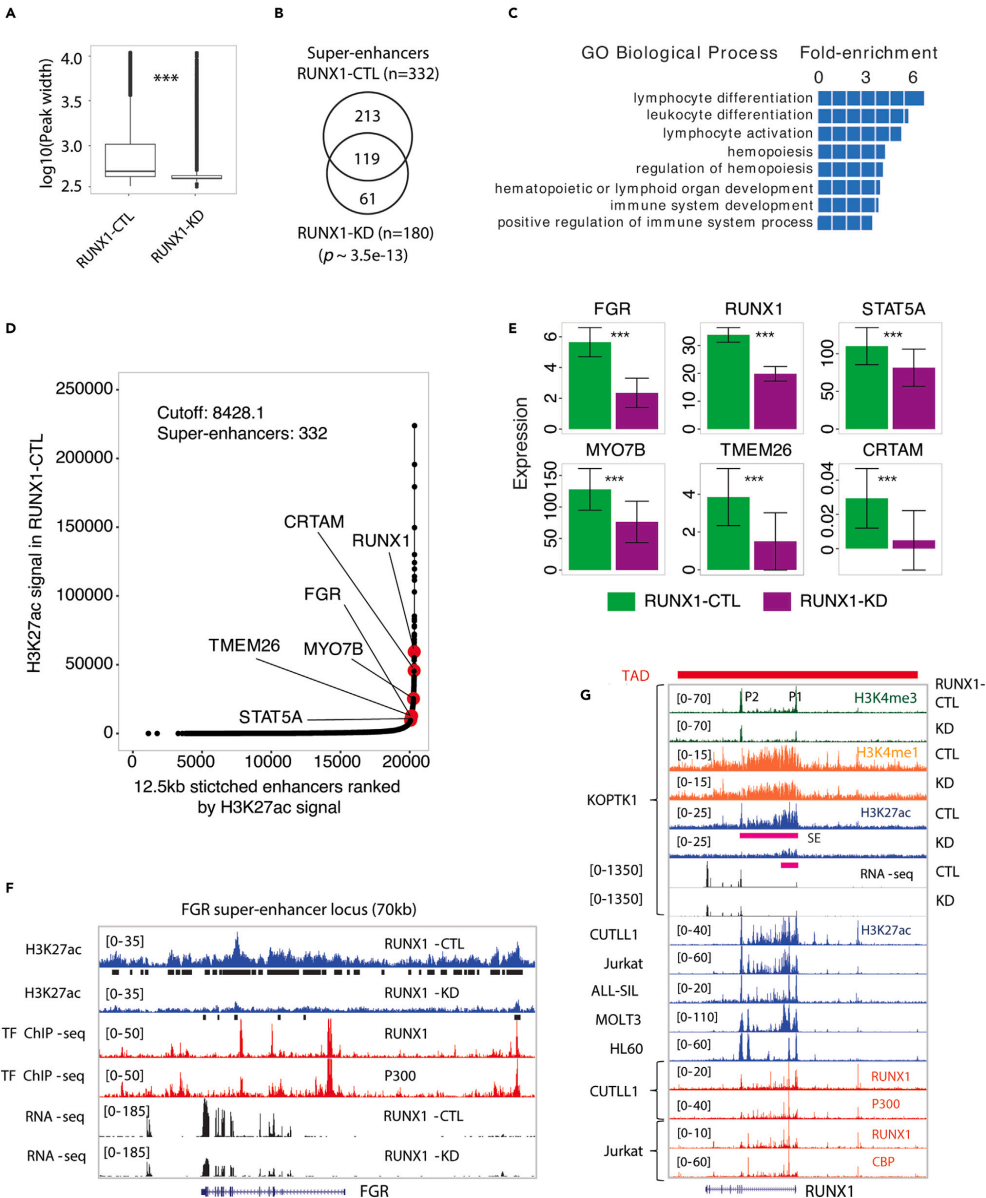
RUNX1 is required for the maintenance of oncogenic super-enhancers (A) Comparison of H3K27ac peak width between RUNX1-CTL and RUNX1-KD samples. An unpaired two-tailed t-test was used to calculate the p value where ∗∗∗ indicates p value is < 0.001. (B) Overlap of number of super-enhancers between RUNX1-CTL and RUNX1-KD samples. Statistical significance was estimated using Fisher’s exact test. (C) Pathway analysis of the gene set nearest to H3K27ac peaks that were within super-enhancers and lost following RUNX1-KD. Top ranked Gene Ontology (GO) biological processes are shown at binomial FDR ∼0. (D) Plot of H3K27ac ChIP-seq signal for stitched peaks in the RUNX1-CTL sample. The key proto-oncogenes that were down-regulated (FDR ≤ 0.05) and showed loss of H3K27ac peaks within super-enhancers upon RUNX1-KD are highlighted. (E) Down-regulation of proto-oncogenes following RUNX1-KD in three independent RNA-seq experiments in KOPTK1 cells. Data are mean ± s.e.m. of reads per kilobase million (RPKM) values and ∗∗∗ indicates FDR ≤ 0.05. (F) Normalized density tracks for H3K27ac, RUNX1, P300, and RNA-seq at the FGR super-enhancer locus. H3K27ac peaks are shown in black bars. Transcription factor (TF) ChIP-seq data were derived from the CUTLL1 T-ALL line. (G) Normalized density tracks for H3K4me3, H3K27ac, H3K4me1, RNA-seq, RUNX1, CBP, P300 ChIP-seq experiments across T-ALL and AML cell lines at the topologically associating domain (TAD) surrounding *RUNX1*. The super-enhancer (SE) region is shown in the pink bar.

Examination of the pattern of H3K27ac loss following RUNX1-KD in KOPTK1 revealed a contraction of H3K27ac density within the majority of peaks (Figure 2A). The combination of contraction and concomitant peak loss of H3K27ac following RUNX1-KD was associated with a 46% reduction in the total number of super-enhancers (Figure 2B). Super-enhancers were more sensitive to RUNX1-KD compared to typical-enhancers (Figure S3E). Upon RUNX1-KD, 70% of the super-enhancer associated H3K27ac peaks showed ≥2-fold reduction of read density whereas there was 47% loss at classical enhancers. Genomic Regions Enrichment of Annotations Tool (GREAT)^33^ analysis of genes proximal to the depleted super-enhancers showed enrichment in lymphocyte activation and differentiation pathways (Binomial False Discovery Rate, FDR = 0) (Figure 2C). These included a set of known and suspected proto-oncogenic drivers (e.g., *FGR, RUNX1, STAT5A, MYO7B, TMEM26,* and *CRTAM*) in T-ALL which lost H3K27ac peaks at the adjacent super-enhancers and were down-regulated (FDR ≤ 0.05) following RUNX1-KD (Figures 2D and 2E). FGR is a member of the Src Family Tyrosine Kinase which is highly expressed in a significant proportion of AML patients and associated with poor prognosis.^34^^,^^35^ We observed an 80% overall reduction in H3K27ac peaks (25/31) within a 70 kb window encompassing the *FGR* super-enhancer and *FGR* expression was significantly (FDR = 0.0009) down-regulated following RUNX1-KD (Figures 2E and 2F).

RUNX1 itself drives an auto-regulatory feedforward circuit with other key hematopoietic transcription factors including GATA3, TAL1, and MYB in T-ALL.^25^^,^^36^ Consistent with this we observed a 144.5 kb super-enhancer within the first intron of RUNX1 that is conserved across T-ALL and AML cell lines and contains 15 binding sites for RUNX1 (Figure 2G). RUNX1-KD reduced H3K27ac levels 2.5-fold within the RUNX1 super-enhancer and reduced H3K4me3 density at both RUNX1 promoters. Collectively, these results suggest that RUNX1 plays an essential role in maintaining super-enhancers and the expression of proto-oncogenic targets of RUNX1 is sensitive to the loss of RUNX1-dependent super-enhancers.

### RUNX1 and NOTCH1 cooperate to establish active promoter states

**Figure 3:**
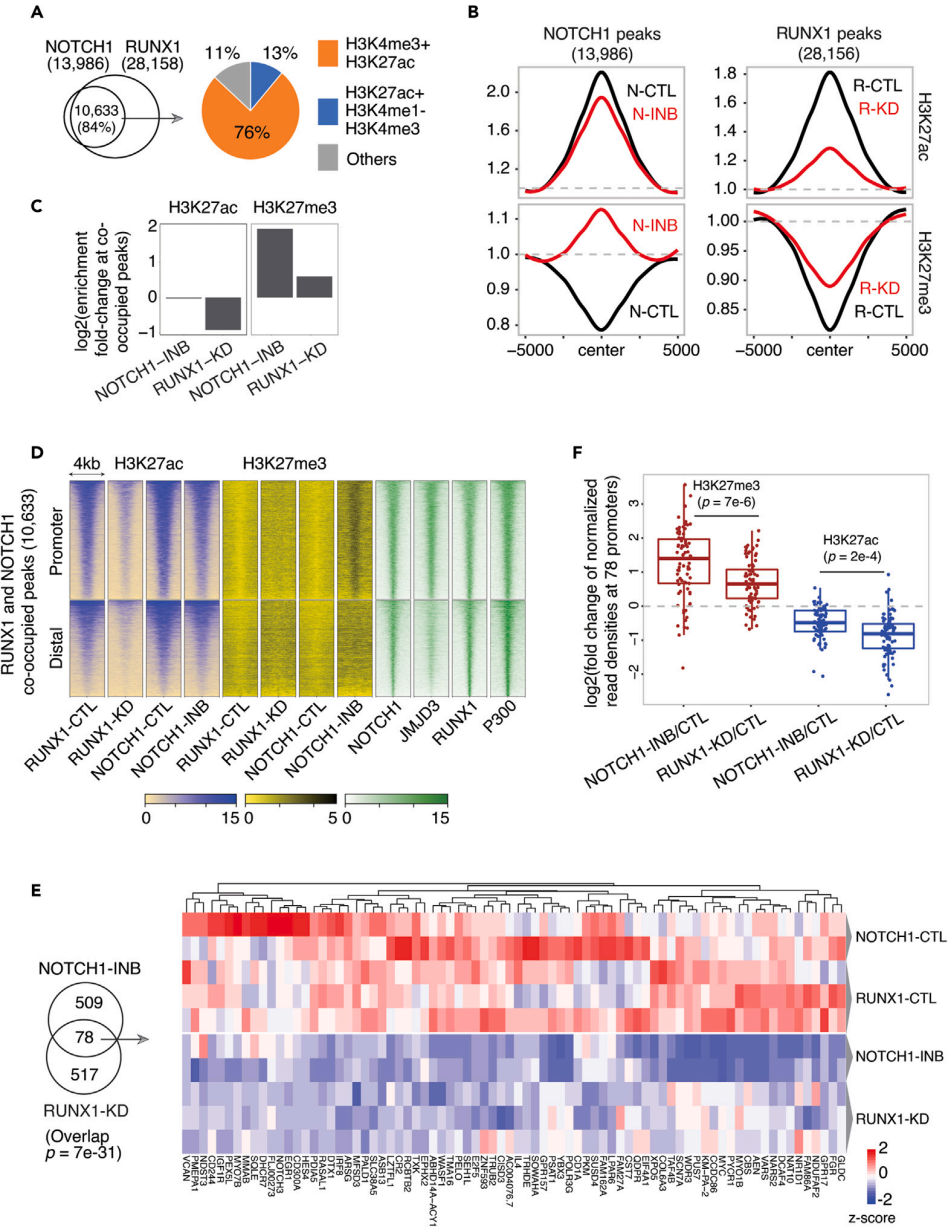
Cooperation between RUNX1 and NOTCH1 to modify H3K27 residues (A) Intersection of RUNX1 and NOTCH1 peaks and their distribution at the active promoter (H3K4me3+H3K27ac) and enhancer (H3K27ac + H3K4me1-H3K4me3) regions defined by the presence of the indicated histone marks. (B) Mean coverage for H3K27ac and H3K27me3 upon RUNX1-KD and NOTCH1-INB around RUNX1 and NOTCH1 peaks, respectively. “N” means NOTCH1 and “R” means RUNX1. Signal intensity at the 5′end of the region was scaled to 1. (C) Fold enrichment of H3K27ac and H3K27me3 at RUNX1 and NOTCH1 co-bound peaks following RUNX1-KD or NOTCH1-INB. (D) Enrichment heatmap for H3K27ac and H3K27me3 following RUNX1-KD or NOTCH1-INB at RUNX1 and NOTCH1 co-occupied peaks in KOPTK1 cells. NOTCH1, JMJD3, RUNX1, and P300 ChIP-seq data were obtained from CUTLL1 T-ALL cell line. Rows representing genomic intervals are sorted by the descending signal of the first column (RUNX1-CTL). (E) Normalized expression (RPKM) of downregulated genes (FDR ≤ 0.05) following NOTCH1-INB or RUNX1-KD. Scaled RPKM values are shown in the heatmap in two replicates of NOTCH1-INB and three replicates of RUNX1-KD experiments in KOPTK1 cells. (F) For H3K27me3 and H3K27ac, fold-change of normalized read densities at the promoters (+-2 kb of transcription start site) of the 78 co-regulated genes following NOTCH1-INB or RUNX1-KD. An unpaired two-tailed t-test was used to calculate the p value. In this figure, TF ChIP-seq data for NOTCH1, RUNX1, JMJD3, and P300 were derived from the CUTLL1 T-ALL line.

NOTCH1-mediated JMJD3 recruitment is essential for leukemic growth by modulating H3K27me3 at the promoters of proto-oncogenes.^26^ NOTCH1 peaks are frequently co-occupied by RUNX1 peaks and RUNX1 is required for the expression of key NOTCH1 target genes in T-ALL models.^11^^,^^19^ Based on these observations, we hypothesized that at the co-occupied peaks, NOTCH1 demethylates H3K27me3 through JMJD3 allowing RUNX1 to acetylate the demethylated H3K27 residue via recruitment of CBP/P300. To test this directly, we first examined the relationship of 28,156 RUNX1 and 13,986 NOTCH1 peaks determined by ChIP-seq in the CUTLL1 T-ALL cell line.^19^ The majority (84%) of NOTCH1 peaks intersected with RUNX1 peaks and 76% of the co-occupied peaks were marked by active promoter modifications (H3K4me3 and H3K27ac) (Figure 3A). RUNX1-KD led to a significant reduction in H3K27ac density around RUNX1 peaks whereas NOTCH1-INB led to an increase in H3K27me3 around NOTCH1 peaks (Figures 3B and S4D), supporting a model of NOTCH1 dependent demethylation of H3K27me3.^26^^,^^27^ At RUNX1 and NOTCH1 co-occupied peaks, RUNX1-KD reduced H3K27ac enrichment by 2-fold and NOTCH1 inhibition increased the H3K27me3 enrichment by 4-fold (Figure 3C). The co-occupied peaks intersecting with promoters had relatively higher enrichment of NOTCH1 and JMJD3 compared to the distal regions and the gain of H3K27me3 following NOTCH1-INB was specifically observed at gene promoters (Figures 3D and S4A). Thus, our analysis supports a cooperative model for NOTCH1 and RUNX1, where NOTCH1 drives the demethylation of H3K27me3 through JMJD3 recruitment to promoters of its target genes allowing for RUNX1-dependent deposition of H3K27ac and establishment of an active chromatin state.

**Figure 4:**
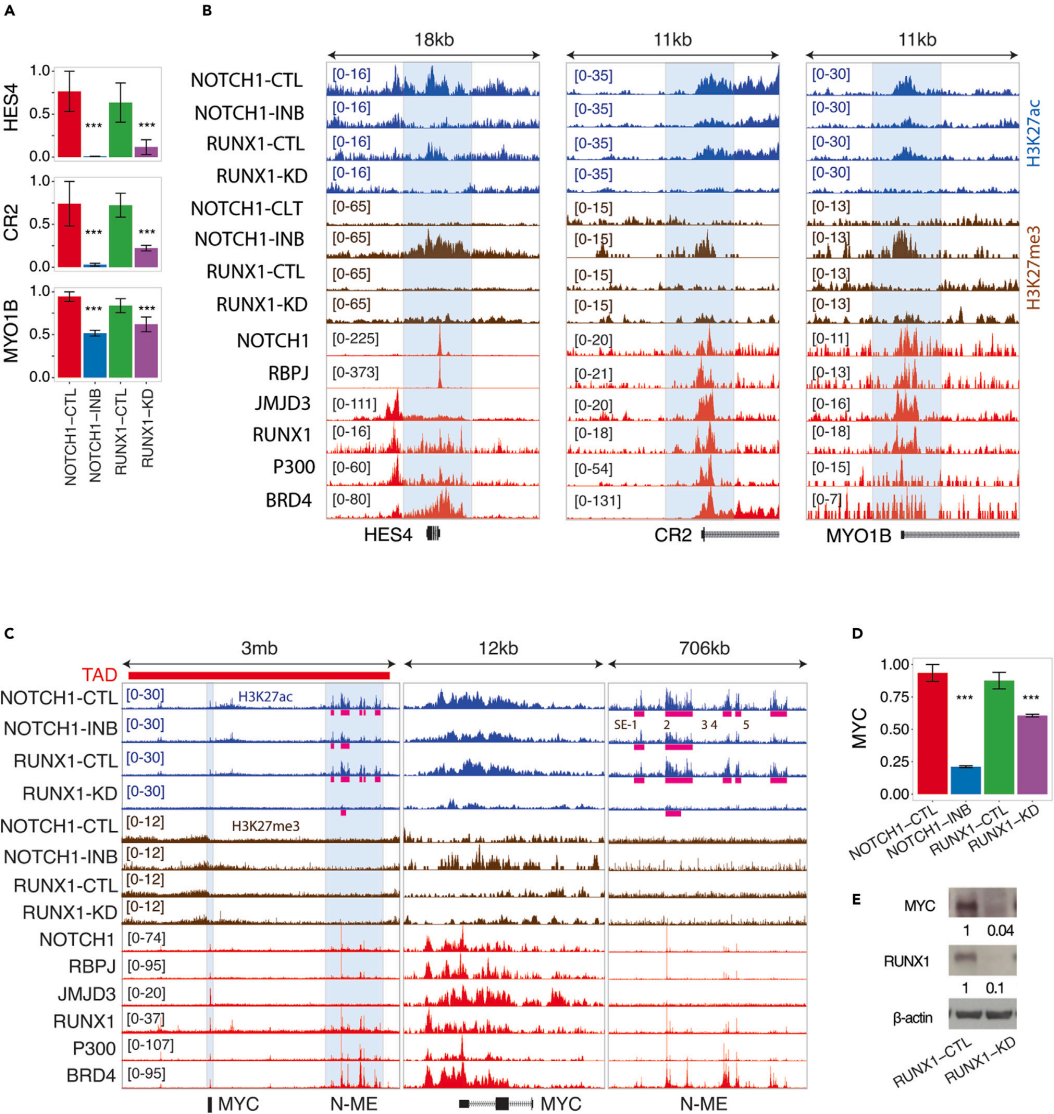
Cooperative transcriptional regulation by RUNX1 and NOTCH1 (A) Relative expression changes following NOTCH1-INB and RUNX1-KD compared to control samples in KOPTK1 cells. Expression of *HES4, CR2,* and *MYO1B* are shown in two replicates of NOTCH1-INB and three replicates of RUNX1-KD. Data are mean ± s.e.m. of scaled RPKM values and ∗∗∗ represents FDR ≤ 0.05 in differential gene expression analysis following RUNX1-KD or NOTCH1-INB. (B) Normalized tracks for H3K27ac and H3K27me3 at the *HES4, CR2,* and *MYO1B* regulatory loci in KOPTK1. NOTCH1, RBPJ, JMJD3, RUNX1, P300, and BRD4 ChIP-seq data were obtained from CUTLL1 T-ALL cell line. (C) Normalized H3K27ac and H3K27me3 tracks at the topologically associating domain (TAD) locus surrounding the *MYC* promoter and enhancers. Five super-enhancers (SE-1 to -5) at N-ME are highlighted in pink bars. (D) Relative expression changes of *MYC* following NOTCH1-INB and RUNX1-KD. Data are mean ± s.e.m. of scaled RPKM values and ∗∗∗ represents FDR ≤ 0.05 in differential gene expression analysis following RUNX1-KD or NOTCH1-INB. (E) Western blot analysis of RUNX1 and MYC protein level following RUNX1-KD. Numbers below the MYC and RUNX1 panels indicate their relative expression level after normalization to β-actin loading control. In this figure, TF ChIP-seq data for NOTCH1, RBPJ, RUNX1, JMJD3, BRD4, and P300 were derived from the CUTLL1 T-ALL line.

Consistent with the cooperative model, 78 genes (hypergeometric p value ∼0) were significantly (FDR ≤0.05) downregulated by either RUNX1-KD or NOTCH1-INB in KOPTK1 cells (Figure 3E and Table S2). The promoters of these 78 genes were significantly depleted of H3K27ac upon RUNX1-KD and gained H3K27me3 upon NOTCH1-INB (Figure 3F). Collectively these 78 genes represent a comprehensive set of RUNX1 and NOTCH1 co-regulated genes in KOPTK1. This set includes previously identified (e.g., *MYC, FGR, IGF1R, DTX1, HES4, NOTCH3, MYO1B, IL7R, CR2, RASAL1,* etc.) and new candidates (e.g., *IRF8, EGR1, IL4, CD1A,* etc.) of RUNX1 and NOTCH1 co-regulated genes (Figures 4A, 4B, S4B, and S4C). Taken together, our analysis supports a model of synergy between RUNX1 and NOTCH1 in the conversion of H3K27 from a repressive (H3K27me3) to an active state (H3K27ac) within the promoters of NOTCH1 target genes (Figure S5).

### RUNX1 regulates the Notch-dependent *MYC* enhancer element (N-ME)

In T-ALL, the oncogenic activity of NOTCH1 is dependent on *MYC* upregulation.^37^ A cluster of enhancers named the Notch-dependent *MYC* enhancer element (N-ME) influences the expression of *MYC* in T-ALL through long-range enhancer-promoter interaction.^38^^,^^39^ N-ME is composed of five super-enhancers with an average length of 50 kb within a span of 706 kb (Figure 4C). NOTCH1 and RUNX1 both co-occupy *MYC* promoter and distal N-ME super-enhancers that were originally proposed as NOTCH1-dependent enhancers.^38^^,^^39^ In support of previous findings, we observed a modest (1.8-fold on average) reduction in H3K27ac density at the super-enhancers embedded within the N-ME following NOTCH1 inhibition in KOPTK1 cells. However, this reduction was not evenly distributed across the N-ME despite NOTCH1 binding, with three out of five super-enhancers (e.g., SE-3, -4, and -5) disrupted and the largest super-enhancer (e.g., SE-2 of 97 kb) unchanged following NOTCH1 inhibition. In contrast, RUNX1-KD led to a more pronounced (3.1-fold on average) H3K27ac loss at the N-ME super-enhancers and disrupted all super-enhancers within N-ME. Loss of H3K27ac density at the *MYC* promoter and disruption of N-ME super-enhancers following RUNX1-KD were associated with a significant down-regulation of *MYC* at both transcript and protein levels in KOPTK1 (Figures 4C–4E). H3K27me3 density increased following NOTCH1 inhibition at the *MYC* promoter and was associated with a down-regulation of *MYC* transcription (Figures 4C and 4D). Thus, our analysis supports a model where the combination of NOTCH1 and RUNX1 is required for chromatin restructuring at the *MYC* regulatory locus controlling *MYC* expression.

### RUNX1 driven super-enhancers are sensitive to I-BET151 treatment

Genes associated with super-enhancers are sensitive to perturbations of key components of the transcriptional machinery and chromatin modifiers including BET proteins occupying super-enhancers.^28^^,^^40^ The ability of oncogenic RUNX1 to establish super-enhancers raised the possibility of specifically targeting the RUNX1 driven super-enhancers using BET inhibitors. To test this directly, we used a pan-BET inhibitor (I-BET151) that has been shown to inhibit the expression of super-enhancer-associated genes in leukemia^41^^,^^42^ and to deplete H3K27ac at the promoters of proinflammatory cytokines induced by β-glucan in monocytes.^43^

**Figure 5:**
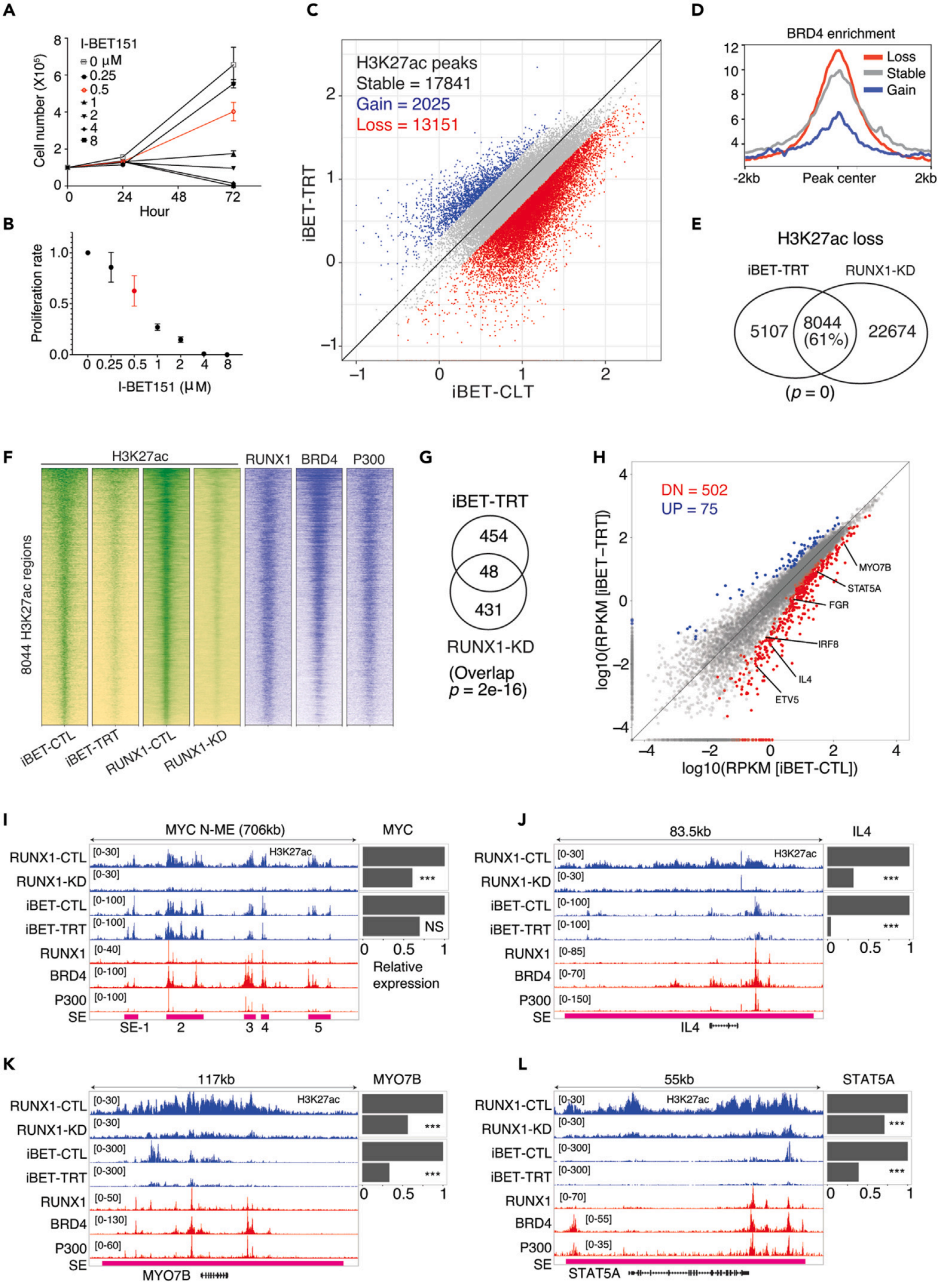
I-BET151 treatment blocks RUNX1 driven super-enhancers (A and B) Cell count and proliferation rates following I-BET151 treatment at four different time points in KOPTK1 cells. Cell count and viability were determined by trypan blue exclusion assay at 72 h of treatment. Experiments were performed in triplicates. (C) Scatterplot using HMD score at H3K27ac peaks between DMSO control (iBET-CTL) and I-BET151 treated (iBET-TRT) samples. The red point indicates ≥ 2-fold loss, blue indicates ≥2-fold gain, and gray indicates stable peaks following I-BET151 treatment. (D) Mean coverage profile for BRD4 at the H3K27ac peaks that are lost, gained, or remained stable following I-BET151 treatment. (E) Overlap of the H3K27ac peaks that were lost following I-BET151 treatment or RUNX1-KD at 2-fold change cutoff. Statistical significance was estimated using Fisher’s exact test. (F) Enrichment heatmap for H3K27ac, RUNX1, BRD4, and P300 at the H3K27ac peaks that were lost following I-BET151 treatment and RUNX1-KD. Rows were sorted by the descending signal of the first column (iBET-CTL). (G) Overlap of the down-regulated genes following I-BET151 treatment or RUNX1-KD. Statistical significance was estimated using Fisher’s exact test. (H) Differential gene expression analysis following I-BET151 treatment. Red points (n = 502) indicate down-regulated, blue points (n = 75) are up-regulated genes and the genes in gray do not show significant change at an FDR cutoff of 0.05. Few of the super-enhancer associated genes are highlighted. (I–L) Genome browser tracks for normalized H3K27ac, RUNX1, BRD4, and P300 around *MYC* N-ME, *IL4, MYO7B,* and *STAT5A* loci. The super-enhancer (SE) regions are shown in the pink bars. In the expression barplot, ∗∗∗ represents FDR ≤ 0.05 in differential gene expression analysis following RUNX1-KD or I-BET151 treatment. NS means not significant. TF ChIP-seq data for RUNX1, BRD4, and P300 were derived from the CUTLL1 T-ALL line.

In KOPTK1 cells, I-BET151 treatment (iBET-TRT) arrested cellular growth and proliferation in a dose-dependent manner (Figures 5A and 5B) consistent with previous studies.^41^^,^^42^^,^^44^ To perform H3K27ac ChIP-seq and RNA-seq, we used 0.5 μM of I-BET151 at which 50% of the KOPTK1 cells showed growth inhibition at 72 h of treatment. We used synthetic DNA-barcoded nucleosomes bearing distinct post-translational modifications as spike-in controls to measure antibody specificity and normalize histone modification density (HMD) at H3K27ac peaks. Analysis of the resulting ChIP-seq datasets revealed a broad reduction of H3K27ac enrichment following I-BET151 treatment where 13151 (40%) peaks reduced HMD ≥ 2-fold (Figure 5C). H3K27ac peaks that were lost following I-BET151 treatment showed higher enrichment of BRD4 compared to the gained or stable H3K27ac peaks, suggesting those depleted H3K27ac peaks have a higher dependency on BRD4 and thus would be expected to be more sensitive to I-BET151 treatment (Figure 5D). I-BET151 treatment displaces the BET proteins BRD2, BRD3, and BRD4 from chromatin.^44^ BRD4 partners with P300 to promote histone acetylation^45^ and P300 also facilitates BRD4 recruitment.^46^ Thus, we hypothesize that in KOPTK1 cells I-BET151 mediated displacement of BET proteins disrupted the interaction with CBP/P300 and reduced H3K27ac HMD. A significant portion (61% (8,044/13,151); Fisher’s exact test; p value ∼0) of depleted H3K27ac peaks following I-BET151 overlapped with RUNX1-dependent peaks suggesting RUNX1-dependent H3K27ac peaks are preferentially affected by I-BET151 treatment (Figure 5E). The overlapping co-dependent H3K27ac peaks (n = 8044) show relatively higher enrichment of BRD4 and H3K27ac than the peaks lost uniquely by RUNX1-KD or I-BET151 treatment, and are co-occupied with RUNX1, BRD4, and P300 (Figures 5F, S6A and S6B). This suggests that the cooperative activities of RUNX1, BRD4, and P300 are facilitating the increased acetylation of H3K27 residues making them sensitive to either RUNX1-KD or I-BET151 treatment. In support of the co-regulation of H3K27ac by RUNX1 and BET proteins, transcriptional programs altered between RUNX1-KD and I-BET151 treatment showed a significant overlap of 48 genes (Fisher’s exact test; p value = 2e-16) including *STAT5A, IL4, IRF8, MYO7B, ETV5,* and *FGR*. (Figure 5G).

RUNX1 and BRD4 peaks more frequently co-occur within super-enhancers than the regions outside of super-enhancers (Figure S6C) and super-enhancers showed reduced H3K27ac density following RUNX1-KD or I-BET151 treatment (Figure S6D). Following I-BET151 treatment, super-enhancers associated proto-oncogenes (e.g., *STAT5A, IL4, ETV6, BCL11A, CD79A, CDK6, ERG,* and *FGR*) known to be involved in leukemogenesis and disease progression, were down-regulated (Figure 5H).^35^^,^^47^^,^^48^^,^^49^^,^^50^^,^^51^^,^^52^^,^^53^ A significant number (18/78; Fisher’s exact test; p value = 5e-13) of RUNX1 and NOTCH1 co-regulated genes were also down-regulated following I-BET151 treatment including super-enhancer associated genes (e.g., *MYC, MYO7B, IL4,* and *FGR*) (Table S3). *MYC* is a known target of bromodomain inhibitors but its sensitivity to such inhibitors varies widely.^42^^,^^54^ At N-ME, only SE-5 was depleted following I-BET151 treatment in KOPTK1 and there was a modest reduction (31% reduction, FDR = 0.38) in *MYC* mRNA levels whereas RUNX1-KD depleted all SEs and *MYC* was significantly down-regulated (40% reduction; FDR = 8e-06) (Figure 5I). *STAT5A, MYO7B,* and *IL4* are associated with super-enhancers and showed marked depletion of H3K27ac densities following RUNX1-KD or I-BET151 treatment and were significantly down-regulated (FDR ≤ 0.05) (Figures 5J–5L). Notably, *MYC*, *STAT5A, MYO7B,* and *IL4* super-enhancers were co-occupied with RUNX1, P300 and BRD4 peaks. Collectively, the results suggest that RUNX1 and BET co-dependent H3K27ac peaks have increased sensitivity to BET perturbation and thus are attractive targets to block the transcription of associated proto-oncogenes.

### RUNX1 drives the expression of *CDC25A* and promotes entry into S-phase of cell cycle

**Figure 6:**
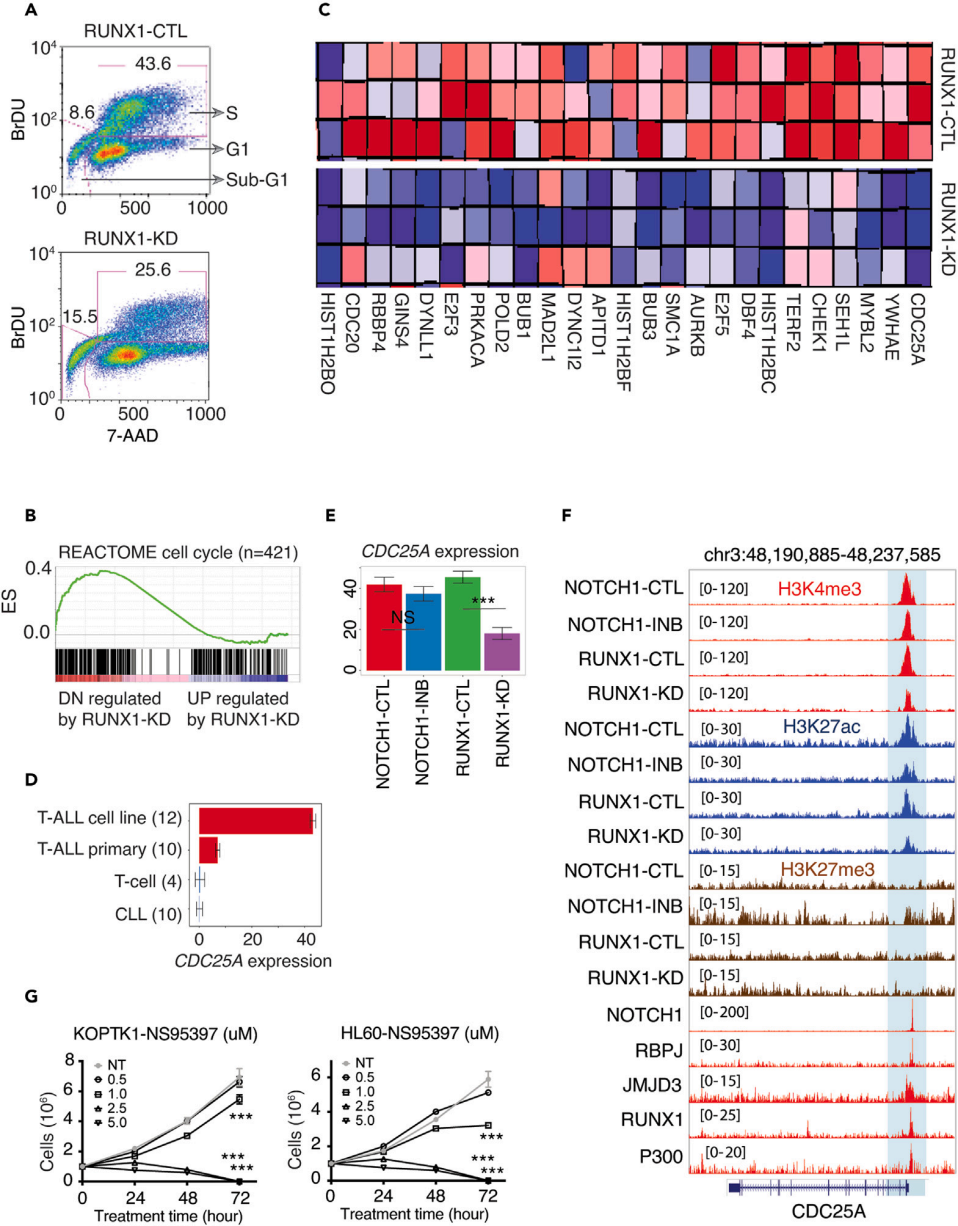
RUNX1 contributes to overcome G1-S cell cycle checkpoint barriers (A) BrdU incorporation assay in KOPTK1 cells at day 7 post-transduction of lentivirus. (B) GSEA using the “REACTOME Cell Cycle genes” as a gene set to estimate the enrichment of genes between RUNX1-CTL and RUNX1-KD samples in RUNX1 knockdown experiments. ES means enrichment score. (C) Heatmap of the top 25 cell cycle genes that showed core enrichment in GSEA between RUNX1-CTL and RUNX1-KD in KOPTK1. Genes are ranked by GSEA rank order from right to left. (D) Expression of *CDC25A* in different cell types. T cells include CD4 naive, CD8 naive, CD4 memory, fetal thymus, and CLL indicates primary chronic lymphocytic leukemia patient samples. Data are mean ± s.e.m. of RPKM values. (E) Expression of *CDC25A* upon RUNX1-KD (3 replicates) and NOTCH1-INB (2 replicates) in KOPTK1. Data are mean ± s.e.m. of RPKM values and ∗∗∗ represents FDR ≤ 0.05 in differential gene expression analysis following RUNX1-KD or NOTCH1-INB. NS means not significant. (F) Genome browser tracks for normalized H3K4me3, H3K27ac, H3K27me3, RUNX1, NOTCH1, PBPJ, JMJD3, and P300 around *CDC25A* locus. TF ChIP-seq data for RUNX1, NOTCH1, PBPJ, JMJD3, and P300 were derived from the CUTLL1 T-ALL line. (G) Live cells were counted in triplicates upon treatment with CDC25A inhibitor (NS95397) at four different concentrations and time points in KOPTK1 and HL60 cell lines. Three independent experiments were performed to calculate p values at 72 h using unpaired two-tailed t-test between non-treated (NT) and NS95397 treated samples at 0.5, 1, 2.5, and 5 μM concentrations. ∗∗∗ indicates p value is < 0.001.

RUNX1 stimulates cell cycle entry and progression in hematopoietic cells^55^^,^^56^ and its knockdown is associated with impaired cell growth, increased apoptosis, and G1-phase accumulation in T-ALL cell lines.^11^^,^^12^^,^^25^ We confirmed the reported block of S-phase with a 44%–26% drop in the proportion of cells in S-phase following RUNX1-KD in KOPTK1 cells (Figure 6A). RUNX1 itself is not a part of the cell cycle pathway but its association with a blocked G1-S transition suggests that RUNX1 might regulate key cell cycle genes. To identify putative RUNX1 target genes involved in cell cycle regulation, we used gene set enrichment analysis (GSEA) with the REACTOME cell cycle gene set (Figures 6B and 6C). The highest-ranked gene in GSEA was *CDC25A* whose over-expression is associated with a shortened G1-phase and faster S-phase entry in the cell cycle.^57^^,^^58^ *CDC25A* is highly expressed in T-ALL (Figure 6D) and its expression was significantly (FDR ≤0.05) down-regulated (>2-fold) upon RUNX1-KD and with a concomitant loss of H3K4me3 (2.17-fold) and H3K27ac (1.65-fold) promoter density (Figures 6E and 6F). NOTCH1 also binds to the *CDC25A* promoter but NOTCH1 inhibition did not alter nearby chromatin states nor transcription significantly. Chemical inhibition of CDC25A by NS95397 in KOPTK1 and HL60 cell lines led to reduced cell viability (t-test p-value < 0.001 at 72 h in triplicates) and phenocopied a RUNX1-KD mediated block of cell growth (Figure 6G). Thus, our data suggest that induction of *CDC25A* by RUNX1-mediated chromatin remodeling contributes to a disruption of the G1-S checkpoint to promote entry into the S-phase in T-ALL cells.

## Discussion

While RUNX1 was initially described as a tumor suppressor in T-ALL,^8^ recent studies in human and mouse T-ALL models suggest it plays a dual oncogenic role.^11^^,^^12^ A tumor-suppressor role for RUNX1 was proposed based on observed loss of function mutations to *RUNX1* in T-ALL.^59^ *RUNX1* mutations are highly prevalent in the early T-cell precursor (ETP) T-ALL and are found in 20% of T-ALL cases.^8^ In contrast to this model, we report here that wild-type RUNX1 binds to DNA elements controlling proto-oncogene expression where it facilitates H3K27ac deposition and gene activation. A RUNX1-dependent increase of H3K27ac establishes oncogenic super-enhancers that drive expression of proto-oncogenes known to be associated with T-ALL pathogenesis including Notch signaling and cell cycle pathways. Taken together, our data support a parallel role for RUNX1 as an oncogene in T-ALL.

RUNX1 and NOTCH1 cooperation has been proposed through the analysis of co-dependent transcriptional changes at selected target genes.^11^^,^^19^ Through the integrative analysis of chromatin and transcriptional states, we now propose a synergistic RUNX1 and NOTCH1 model that acts in a stepwise fashion to move chromatin from a repressed to an active state and have cataloged a comprehensive list of shared target genes. The synergistic relationship of NOTCH1 and RUNX1 extends to super-enhancers in T-ALL which were previously reported to be regulated by NOTCH1.^19^ Here we show that RUNX1 is required for the maintenance of super-enhancers including N-ME and thus the previously reported dependence on NOTCH1 may lie in the requirement for an initial demethylation of H3K27me3 through JMJD3 recruitment. More generally, RUNX1 and NOTCH1 co-regulated genes were highly sensitive to either RUNX1-KD or NOTCH1 inhibition, suggesting that NOTCH1 and RUNX1 both contribute to forming a transcriptional regulatory unit that can be disrupted by the inhibition of either partner. In support of this, RUNX1 has been described as part of the ICN1 interactome^60^ setting the stage for future work to identify the basis and extent of physical interactions between ICN1 and RUNX1 interactomes at co-regulated loci. Combining previous observations^26^^,^^27^ along with those revealed in this study, we suggest that one mechanism for RUNX1 and NOTCH1 co-regulation of their target loci involves a stepwise activation of H3K27me3 marked regions with NOTCH1 mediating the removal of the repressive trimethyl modification followed by RUNX1 mediated acquisition of H3K27ac. However, this model does not refute the hypothesis that RUNX1 might be required to acetylate the enhancers first to recruit RBPJ/NOTCH1 complex at some loci which has been previously shown in *Drosophila*.^20^

Previous studies in T-ALL implicate multiple RUNX family members, particularly RUNX1 and RUNX3, on the basis that they are frequently co-expressed and show functional redundancies.^11^ However, RUNX1 is expressed across all T-ALL subtypes and forms a core regulatory circuitry with TAL1, GATA3, and MYB, driving the growth and survival of T-ALL cells.^11^^,^^12^^,^^25^^,^^36^ Our data on leukemia and epithelial cancer cell lines showed RUNX1 regulates global H3K27ac levels and thus has the potential to co-bind with other oncogenic factors at H3K27ac marked enhancers. In support of this, a recent study suggests RUNX1 plays a role in regulating global H3K27ac levels through cooperation with key transcription factors in limbal stem cells.^61^ It will be of interest to determine if the cooperative role of RUNX1 with oncogenic (co)factors in T-ALL cells extends to other cancers through synergistic partnerships with additional transcription factors.

Deregulation of enhancer states has emerged as a critical step in the activation and maintenance of aberrant transcriptional programs in T-ALL,^36^^,^^38^^,^^62^^,^^63^ which provides rationale for targeting chromatin regulators associated with enhancers as a viable anti-cancer strategy, such as pharmacological inhibition of BET proteins. BET bromodomain inhibitors displace BET proteins from chromatin^28^^,^^41^^,^^44^ and represent promising therapeutic agents in hematopoietic and solid cancers.^64^ The findings presented here suggest that BET inhibition led to a reduction in H3K27ac levels preferentially at the BRD4 enriched regions and disrupted RUNX1 driven super-enhancers, suggesting a therapeutic opportunity to target RUNX1 driven enhancers in T-ALL. Taken together, our study highlights the central role of RUNX1 in establishing aberrant enhancers and provides the mechanistic basis for targeting aberrant enhancers using BET inhibitors in RUNX1-driven malignancies.

### Limitations of the study

In this study, we demonstrated that a reduction in RUNX1 levels correlates with a genome-wide reduction of H3K27ac levels. While we propose a mechanistic model that directly links RUNX1 to this loss, future studies will be required to directly test this and other potential models. Despite an overall reduction in H3K27ac occupancy following RUNX1-KD a limited number of H3K27ac regions including those at super-enhancers demonstrated an increase in H3K27ac density. Our proposed model does not explain the mechanism of gain of H3K27ac following RUNX1-KD but we speculate these gains represent secondary effects of RUNX1-KD.

Our study provides evidence of a synergistic relationship between RUNX1 and NOTCH1 to establish active promoter and enhancer states. This model of synergy is based on a correlation between histone modification states and transcriptomic changes following RUNX1 and NOTCH1 perturbation in KOPTK1 and its generalizability remains to be explored. Furthermore, our analysis does not provide direct evidence that upon NOTCH1 or RUNX1 perturbation the interaction between RUNX1 and the ICN1 complex is disrupted, a prediction of our model. Finally, while we provide evidence that I-BET151 treatment is associated with a broad reduction of H3K27ac levels in KOPTK1, additional BET inhibitors and RUNX1-driven cell lines should be tested to confirm whether BET inhibition alone is sufficient to reduce RUNX1-mediated H3K27ac levels.

## STAR★Methods

### Key resources table

**Table:** 

REAGENT or RESOURCE	SOURCE	IDENTIFIER
**Antibodies**		

Anti-H3K27me3	Diagenode	Cat: C15410195; Clone: poly; Lot: A1811-001P; RRID: AB_2753161
Anti-H3K27ac	Hiroshi	Clone: mouse mAb (CMA309-IgG1)
Anti-H3K4me3	Cell Signaling	Cat: #9751; Clone: C42D8; RRID: AB_2616028
Anti-H3K4me1	Diagenode	Cat: C15410037; Clone: poly; Lot: A1657D
Anti-H3K36me3	Diagenode	Cat: C15410192; Clone: poly; RRID: AB_2744515
Anti-H3K9me3	Diagenode	Cat: C15410056; Clone: poly
Rabbit anti-H3	Sigma	Cat #H9289, 1:1000; RRID: AB_1079063

**Chemicals, peptides, and recombinant proteins**		

pLKO.1 PuroR expression vector	Addgene	1864
Gamma secretase inhibitor (GSI)	EMDMillipore	Cat # 565790
I-BET151	Selleckchem	S2780
DMSO	Sigma	D2650-100 ML
NS95397	TOCRIS	NSC 95397 (Cat # 1547)

**Deposited data**		

Raw and analyzed data	This paper	GSE99706

**Experimental models: Cell lines**		

T-ALL	Jon C. Aster’s lab, Brigham and Women’s Hospital, Boston, MA, USA	KOPTK1^65^
T-ALL	Andrew P. Weng’s lab, BC Cancer, Vancouver, BC V5Z 1L3, Canada	CUTLL1^66^
T-ALL	Connie Eaves’s lab, BC Cancer, Vancouver, Canada	Jurkat
T-ALL	Andrew P. Weng’s lab, BC Cancer, Vancouver, Canada	HPB-ALL^67^
T-ALL	Andrew P. Weng’s lab, BC Cancer, Vancouver, Canada	RPMI 8402^68^
Prostate adenocarcinoma	ATCC (HTB-81™)	DU145
AML	Connie Eaves’s lab, BC Cancer, Vancouver, Canada	HL60^69^

**Software and algorithms**		

BWA (v0.5.7)	Li and Durbin,^70^	https://bio-bwa.sourceforge.net/
FindER (v1.0.0b)	Bilenky and Hirst,^29^	https://thisisepigenetics.ca/data/CEMT/tools/finder1/index.html
BEDTools (v2.29.0)	Quinlan and Hall,^71^	https://bedtools.readthedocs.io/en/latest/
deepTools (v3.3.0)	Ramirez et al.^72^	https://deeptools.readthedocs.io/en/develop/
JAGuaR (v2.0.3)	Butterfield et al.^73^	https://www.bcgsc.ca/resources/software/jaguar
DEfine	Gascard et al.^74^	
CRISPResso	Pinello et al.^75^	http://crispresso2.pinellolab.org
ROSE	Whyte et al.^76^	http://younglab.wi.mit.edu/super_enhancer_code.html

### Resource availability

#### Lead contact

Further information and requests for resources and reagents should be directed to the lead contact, Martin Hirst (hirstm@mail.ubc.ca).

#### Materials availability

The cell lines used in this study are available from the lead contact.

### Experimental model and subject details

#### Cell lines and growth condition

KOPTK1, RPMI, HPBALL, Jurkat and HL60 cells were grown in RPMI 1640 media supplemented with 10% FBS, penicillin/streptomycin solution, 2 mM L-glutamine and 1 mM sodium pyruvate. DU145 cells were grown in EMEM with 10% FBS in 37°C at 5% CO2.

### Method details

#### NOTCH1 inhibition

Cells were treated with 1 μM gamma secretase inhibitor (GSI) or vehicle (DMSO) for 3 days and sorted for viability by FACS prior to sequencing. GSI was obtained from EMDMillipore, Cat # 565790.

#### Lentiviral knockdown of RUNX1

For RUNX1 knockdown by shRNA, cells were transduced with shRUNX1 (e.g., shRUNX1-58, shRUNX1-59, and shRUNX1-90) lentiviruses carrying a puromycin resistance marker. At 5 days post-transduction, cells were harvested for ChIP-seq and RNA-seq. High titer lentivirus was generated by transient co-transfection of 293T cells with packaging envelope vectors using the similar method described previously.^12^^,^^77^ The pLKO.1 PuroR expression vector containing the scrambled non-silencing control was obtained from Addgene (#1864). The RUNX1 targeting shRNAs, e.g., shRUNX1-58 (TRCN0000013658), shRUNX1-59 (TRCN0000013659), and shRUNX1-90 (TRCN0000013660) were obtained and cloned using the similar method described previously.^78^ All constructs were verified by sequencing. Virally transduced cells were isolated by fluorescence activated cell sorting (FACS) or by selection with puromycin as applicable.

#### CRISPR-Cas9 knockout of RUNX1

We generated RUNX1-KO using Alt-R CRISPR-Cas9 genome editing system following the manufacturer’s guideline (Integrated DNA Technologies, Inc.). Ribonucleoprotein complexes (crRNA:tracrRNA duplex) were delivered using Neon Transfection System. For KOPTK1, and HL60, 2×10^5^ cells per 10 μL reaction were electroporated at 1600V, 10 ms, 3 pulses. For Jurkat, 150,000 cells per 10 μL reaction, 1600V, 10 ms, 3 pulses. For DU145, 0.8×10^5^ cells per 10 μL reaction were electroporated at 1250V, 20 ms, 2 pulses. Two separate guide RNAs were used to generate RUNX1-KO1 and RUNX1-KO2 knockout samples for the above mentioned cell lines. After 72 h, amplicons were generated by PCR using the primers targeting RUNX1 cut-sites in the knockout samples. A customized version of the paired-end sample Prep Kit from Illumina V.3. was used for sample preparation. Libraries were indexed, pooled, and sequenced using paired-end 75 nt sequencing chemistry on an Illumina MiSeq platform following the manufacturer’s protocols (Illumina, Hayward, CA). Sequencing data were analyzed by CRISPResso^75^ for the annotation of mutations in RUNX1-KO1 and RUNX1-KO2 knockout samples. The IDT online tool (https://www.idtdna.com/pages/tools/) was used to design the following CRISPR guide RNAs and sequencing primers.

gRNA-KO1 (Design ID: Hs.Cas9.RUNX1.1.AA): CACTTCGACCGACAAACCTG. Location: hg19:chr21:36252859-36252879 = 20bp.

gRNA-KO2 (Design ID: Hs.Cas9.RUNX1.1.AC): GCCATCTGGAACATCCCCTA. Location: hg19:chr21:36252980-36253000 = 20bp.

Primers for sequencing gDNA-KO1: forward: 5′-CACAATTCCTACGTTGCATGTT-3′, reverse: 5′-TTCTTCATGGCTGCGGTAG-3′.

Primers for sequencing gDNA-KO2: forward: 5′-CTAGGGATTCCATCACAGAAATC-3′, reverse: 5′-CAGTCAAAGGACAAATGCAGAC-3′.

#### Global H3K27ac measurements

According to the manufacturer’s protocol, the total histone protein was extracted using Histone Extraction kit (Abcam, Cat # ab113476). Total histone protein was quantified using BCA assay. H3 and H3K27ac histone proteins were analyzed by standard SDS-PAGE in 4%–12% gel of NuPAGE electrophoresis system (Life Technology, Thermo Fisher). The antibodies used here are Rabbit anti-H3 (N-terminal) (Sigma, Cat #H9289, 1:1000), mouse anti-H3K27ac antibody from Dr. Hiroshi Kimura.^79^ Secondary antibodies were goat anti-rabbit IRDye-800CW (LI-COR, 1:10000), goat anti-mouse IRDye-680RD (LI-COR, 1:10000). The blots were imaged by LI-COR Odyssey CLx Infrared Imaging System. ImageJ was used for quantitative analysis of western blot images.^80^

#### BrdU incorporation assay

We assessed the cell cycle progression by incorporating synthetic thymidine analogs such as BrdU. BrdU incorporation assay quantified newly synthesized DNA during replication in the S-phase of the cell cycle. Cells were transduced with GFP-tagged lentiviral shRNAs targeting shRUNX1 or scrambled shRNA control. Cultures were assayed for BrdU incorporation at day 7 post-transduction with DNA content staining by 7-AAD as described in.^12^ After selection with puromycin to eliminate uninfected cells, BrdU incorporation was measured to identify cells entering into the S-phase.

#### Western blot of RUNX1 and MYC

^12^Major steps involved in the western blot experiment are: (1) at first the whole-cell lysates were separated by sodium dodecyl sulfate-polyacrylamide gel electrophoresis, (2) the lysates were then transferred to Hybond-ECL membranes (Amersham), (3) Hybond-ECL membranes were probed with antibodies against RUNX1, β-actin, or MYC along with HRP-conjugated secondary antibodies. Finally, the band intensities were measured using Image Studio Lite (LI-COR) software.

#### ChIP-seq

For RUNX1 knockdown and NOTCH1 inhibition experiments, ChIP-seq libraries were constructed from 1 million cells by crosslinking frozen cell pellets and DNA sonication using Sonic Dismembrator 550. The list of antibodies used is available here https://thisisepigenetics.ca/data/CEMT/metadata/antibody_qc.html. Input DNA libraries were constructed for background correction during peak calling. A customized version of the paired-end sample Prep Kit from Illumina V.1.1 was used for sample preparation. Libraries were indexed, pooled, and sequenced using paired-end 75 nt sequencing chemistry on an Illumina HiSeq 2500 platform following the manufacturer’s protocols (Illumina, Hayward, CA). The resulting sequence reads were split by index and aligned to the reference human genome (GRCh37-lite; http://www.bcgsc.ca/downloads/genomes/9606/hg19/1000genomes/bwa_ind/genome/README.GRCh37-lite) using BWA (v0.5.7) aln.^70^ Additional ChIP-seq datasets^19^^,^^36^^,^^62^^,^^81^ used in this study are listed in Table S4.

#### ChIP-seq data analysis

In all downstream analyses, duplicate reads were removed and mapQ ≥ 5 was used. FindER (v1.0.0b) was used with IP and DNA input control libraries to call the peaks at FDR ≤0.01.^29^ Genomic features were analyzed by BEDTools (v2.29.0).^71^ Super-enhancers were identified using the ROSE algorithm.^76^ ChIP-seq tracks were RPKM normalized using deepTools (v3.3.0) by ignoring duplicate reads with the bin size of 20bp.^72^ The CTCF binding sites and topologically associating domains (TADs) shared by GM12878 lymphoblastoid, Jurkat cells, and K562 CML cells were identified in GSE68978.^62^ Enrichment of genomic regions was calculated using the following formula where genome size in base pair (bp) was used as background.

$$Enrichment=\frac{\frac{Overlap\;in\;bp\;between\;the\;query\;and\;target\;regions}{Genomic\;occupancy\;in\;bp\;of\;query\;regions}}{\frac{Genomic\;occupancy\;in\;bp\;of\;the\;target\;regions}{Genome\;size\;in\;bp}}$$

#### I-BET151 treatment and SNAP-ChIP spike-ins

KOPTK1 cells were seeded at 100,000 per well in 6 well-plate. DMSO (Sigma, D2650-100 ML) and I-BET151 (Selleckchem, S2780) were dosed by direct addition to the culture media at 0.1%. I-BET151 was used at a final concentration of 0.5 μM and cells were treated for 72 h. Cell count and viability were determined by Trypan Blue exclusion assay at 72 h of treatment and pellets were collected and snap-frozen for further analysis. For SNAP-ChIP, barcoded nucleosomes were obtained from EpiCypher. SNAP-ChIP was performed based on the native ChIP protocol describe by Lorzadeh et al.^82^ by adding 1 μL spike-in after digestion. We normalized the H3K27ac libraries following I-BET151 treatment using DNA-barcoded nucleosomes as spike-in control following the protocol described by Grzybowski et al.^83^ To measure histone modification density (HMD) score at H3K27ac peaks, we used the pipeline developed by Grzybowski et al.^83^

#### RNA-seq

RNA was isolated from cells with TRIzol reagent followed by purification over PureLink RNA mini kit columns (Invitrogen). RNA-seq was performed using a poly-A library construction protocol with strand-specific cDNA synthesis and 8 cycles of PCR. Purification of poly-A+ mRNA was performed by flow-through total RNA using MultiMACS 96 Separation Unit. For library construction, 96-well Plate-based Strand-specific cDNA Synthesis using Maxima H Minus Strand Specific 96-well was used for Illumina HiSeq 2500 Sequencing.

#### RNA-seq data analysis

RNA-seq paired-end reads were aligned to an extended reference transcriptome that consists of the reference genome and annotated exon-exon junctions using BWA (v0.5.7) aln.^70^ We used JAGuaR (v2.0.3) pipeline to generate the custom reference transcriptome (built from NCBI GRCh37-lite reference and Ensembl v75 annotations) and reposition reads that spanned exon-exon junctions.^73^ An in-house pipeline was used to generate RNA-seq quality control matrices and profiles to assess the quality of RNA-seq as described.^74^ To quantify the exon and gene expression, we calculated Reads Per Kilobase Million (RPKM) matrix as described.^84^ For RPKM normalization, we used the total number of reads aligned into coding exons normalized by total exon length. We excluded the reads from mitochondrial genome, ribosomal genes, and the reads falling into the top 0.5% expressed exons which were considered as a source of potential outliers. The gene RPKM was calculated by taking the average RPKM values of all exons of a given gene. Pairwise comparisons between control and treatment were performed to identify differentially expressed genes using a custom DEfine MATLAB tool (FDR ≤ 0.05, Minimum number of aligned reads = 10).^74^ The RNA-seq data for CD4 naive, CD8 naive, CD4 memory and fetal thymus were obtained from Roadmap Epigenomics Project^85^ and processed uniformly using our pipeline. Additional RNA-seq datasets^85^^,^^86^^,^^87^ used in this study are listed in Table S4.

#### Gene set enrichment analysis (GSEA)

To find the enrichment of RUNX1-regulated genes to the REACTOME cell cycle genes, we used the RPKM values of protein-coding genes for seven RUNX1 knockdown experiments in KOPTK1, HPBALL, and RPMI cell lines.

### Quantification and statistical analysis

Numerical analyses and statistical tests were performed on R (v3.6.0). Statistical significance was defined by p values or adjusted p values.

The comparisons of the mean RUNX1 ChIP-seq read densities at gained, lost, and stable H3K27ac regions following RUNX1-KD were performed by a Kolmogorov-Smirnov test (Figure 1E).

Three independent experiments were performed to calculate p values using unpaired two-tailed t-test where ∗ indicates p value ≤ 0.05 and ∗∗ for p value ≤ 0.01 (Figure 1G).

The comparison of H3K27ac peak width between RUNX1-CTL and RUNX1-KD samples was performed by an unpaired two-tailed t-test where ∗∗∗ indicates p value is < 0.001 (Figure 2A).

The p value of overlap of number of genomic regions or genes was estimated using Fisher’s exact test (Figures 2B, 3E, 5E, 5G, S2B and S6C).

Down-regulation of genes following RUNX1-KD or NOTCH1-INB in KOPTK1 cells was indicated by ∗∗∗ for FDR ≤ 0.05 using differential gene expression analysis (Figures 2E, 3E, 4A, 4D, 5I, 5L, 6E, S4B).

The comparison of H3K27me3 and H3K27ac fold-change at the promoters of the 78 co-regulated genes following NOTCH1-INB or RUNX1-KD was performed by an unpaired two-tailed t-test to calculate the p value (Figure 3F).

Three independent experiments were performed to calculate p values at 72 h using unpaired two-tailed t-test between NT and NS95397 treated samples at 0.5, 1, 2.5 and 5 μM concentrations. ∗∗∗ indicates p value is < 0.001 (Figure 6G).

For significance, one-way analysis of variance (ANOVA) was used with multiple comparison test; NS–not significant in (Figure S2E).

The comparison of number of live cells in control (RUNX1-WT) and RUNX1-KO samples at 72 h in HL60 and DU145 cell lines was estimated using an unpaired two-tailed t-test. ∗∗∗ is the t-test p value ≤ 0.05 in three independent experiments (Figures S3A and S3B).

The comparison of normalized H3K27ac density within super-enhancers following RUNX1-KD or I-BET151 treatment in KOPTK1 cells was performed using an unpaired two-tailed t-test (Figure S6D).

## Data Availability

• The RNA-seq and ChIP-seq data have been deposited to the Gene Expression Omnibus (GEO) database via accession number GSE99706, which is publicly available. Differentially expressed genes, super-enhancers, gene expression matrices, and sample metadata are added in the Data S1. • This paper does not report original code. • Any additional information required to reanalyze the data reported in this paper are available from the lead contact upon request.

## Supplemental information

Document S1. Figures S1–S6 and Tables S1–S4

Data S1. Processed data and sample metadata, related to Figures 1–6, and the Data and code availability section of STAR Methods
