# Supplementary material for: RUNX1 colludes with NOTCH1 to reprogram chromatin in T cell acute lymphoblastic leukemia
Source: iScience. 2023 May 3;26(6):106795. doi: 10.1016/j.isci.2023.106795 (PMC10199266; doi:10.1016/j.isci.2023.106795)
Supplement: Document S1. Figures S1–S6 and Tables S1–S4 [file mmc1.pdf]

iScience, Volume *26*

Supplemental information

RUNX1 colludes with NOTCH1 to reprogram chromatin in T cell acute lymphoblastic leukemia

Rashedul Islam, Catherine E. Jenkins, Qi Cao, Jasper Wong, Misha Bilenky, Annaïck Carles, Michelle Moksa, Andrew P. Weng, and Martin Hirst

Supplemental information

a

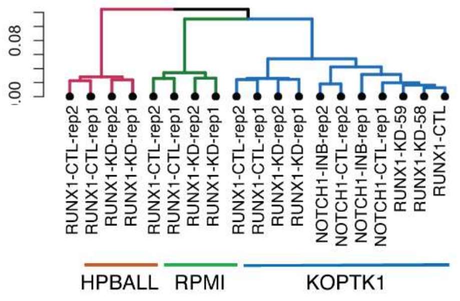

b

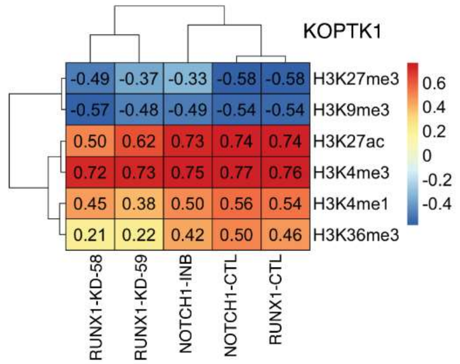

c

STR profiling of KOPTK1 cell line

Cell	D3S1358	TH01	D21S11	D18S51	Penta E	D5S818	D13S317	D7S820
KOPTK1	15, 16	7, 9	29, 32.2	16, 21	5, 12	10, 11	9	10, 12

D16S539	CSF1PO	Penta D	vWA	D8S1179	TPOX	FGA	AMEL	Mouse
9, 11	10, 11	9	14	13, 15	8, 11	20, 22	X, Y	Not Detected

d

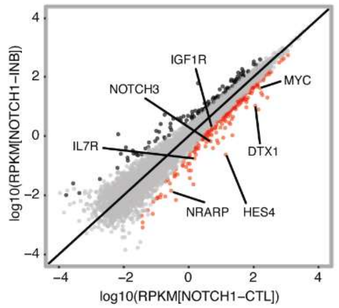

e

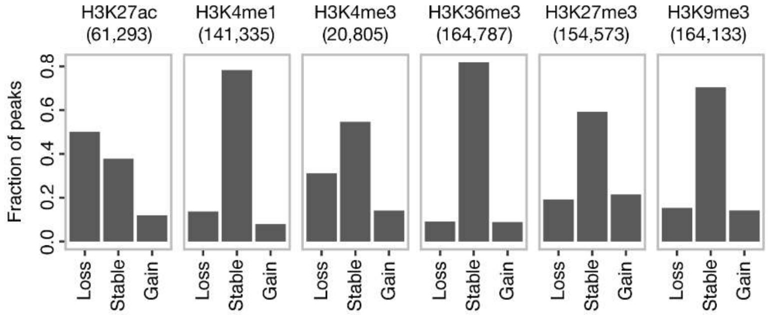

Figure S1: NOTCH1 inhibition and RUNX1 knockdown experiments. Related to Figure 1.

a) Hierarchical clustering of normalized gene expression (RPKM) values of 20,134 protein coding genes. Clustering distance = (1-Spearman correlation) and method = "complete". **b)** Spearman correlation between expression of protein-coding genes and ChIP-seq read densities at promoters for active and repressive histone marks. **c)** Short tandem repeat (STR) profiling for the identity of KOPTK1 cell line. **d)** Differential gene expression analysis following NOTCH1 inhibition with GSI. Red points (n=226) indicate down-regulated, black points (n=127) are up-regulated genes and the genes in grey color do not show significant change at an FDR cutoff of 0.05. **e)** Fraction of ChIP-seq peaks that were lost, gained, or remained stable following RUNX1-KD have been shown for six histone marks. Two fold-change of normalized read density was used to define gain and loss of peaks.

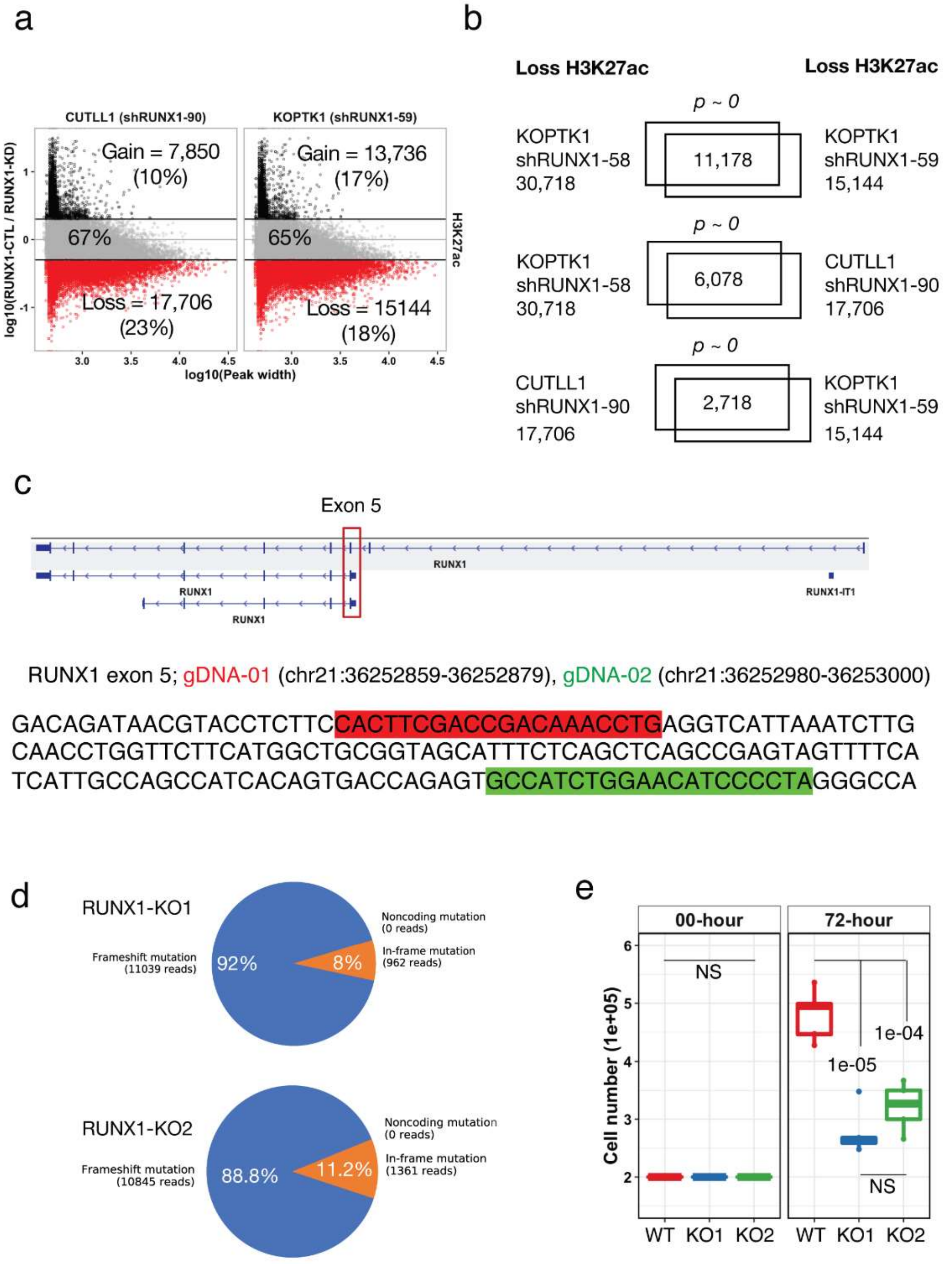
a
CUTLL1 (shRUNX1-90)
KOPTK1 (shRUNX1-59)
Gain = 7,850
(10%)
67%
Loss = 17,706
(23%)
Gain = 13,736
(17%)
65%
Loss = 15144
(18%)
H3K27ac
log10(RUNX1-CTL / RUNX1-KD)
log10(Peak width)
b
Loss H3K27ac
Loss H3K27ac
p ~ 0
KOPTK1
shRUNX1-58
30,718
11,178
KOPTK1
shRUNX1-59
15,144
p ~ 0
KOPTK1
shRUNX1-58
30,718
6,078
CUTLL1
shRUNX1-90
17,706
p ~ 0
CUTLL1
shRUNX1-90
17,706
2,718
KOPTK1
shRUNX1-59
15,144
c
Exon 5
RUNX1
RUNX1
RUNX1
RUNX1-IT1
RUNX1 exon 5; gDNA-01 (chr21:36252859-36252879), gDNA-02 (chr21:36252980-36253000)
GACAGATAACGTACCTCTTCCACTTCGACCGACAAACCTGAGGTCATTAAATCTTG
CAACCTGGTTCTTCATGGCTGCGGTAGCATTTCTCAGCTCAGCCGAGTAGTTTTCA
TCATTGCCAGCCATCACAGTGACCAGAGTGCCATCTGGAACATCCCCTAGGGCCA
d
RUNX1-KO1
Frameshift mutation
(11039 reads)
92%
8%
Noncoding mutation
(0 reads)
In-frame mutation
(962 reads)
RUNX1-KO2
Frameshift mutation
(10845 reads)
88.8%
11.2%
Noncoding mutation
(0 reads)
In-frame mutation
(1361 reads)
e
00-hour
72-hour
Cell number (1e+05)
NS
1e-04
1e-05
NS
WT KO1 KO2
WT KO1 KO2

Figure S2: Role of RUNX1 in H3K27ac regulation. Related to Figure 1.

a) Scatter plot for normalized read densities of H3K27ac upon RUNX1-KD by shRUNX1-90 in CUTLL1 and by shRUNX1-59 in KOPTK1. Each point represents an H3K27ac peak where the red point indicates >= 2-fold loss, black indicates >=2-fold gain and grey indicates stable peaks following RUNX1-KD. Read densities were normalized by sequencing depth. **b)** Overlap of loss of H3K27ac peaks between different shRUNX1s and cell lines. Statistical significance was estimated using Fisher's exact test. **c)** RUNX1 exon-5 (covers DNA binding Runt domain) was targeted to be edited by two gRNAs using the CRISPR-Cas9 system. Exon-5 is common among three major isoforms of RUNX1 and is shown in the red box. **d)** Amplicon sequencing results showing the proportion of reads that have mutations by CRISPR-Cas9 editing in KOPTK1. **e)** Number of cells are shown in boxplot for control (RUNX1-WT) and RUNX1-KOs (e.g., KO1 and KO2) at 00 and 72 hours. Cell numbers from 5 independent experiments are shown. For significance, one-way analysis of variance (ANOVA) was used with multiple comparison test; NS–not significant.

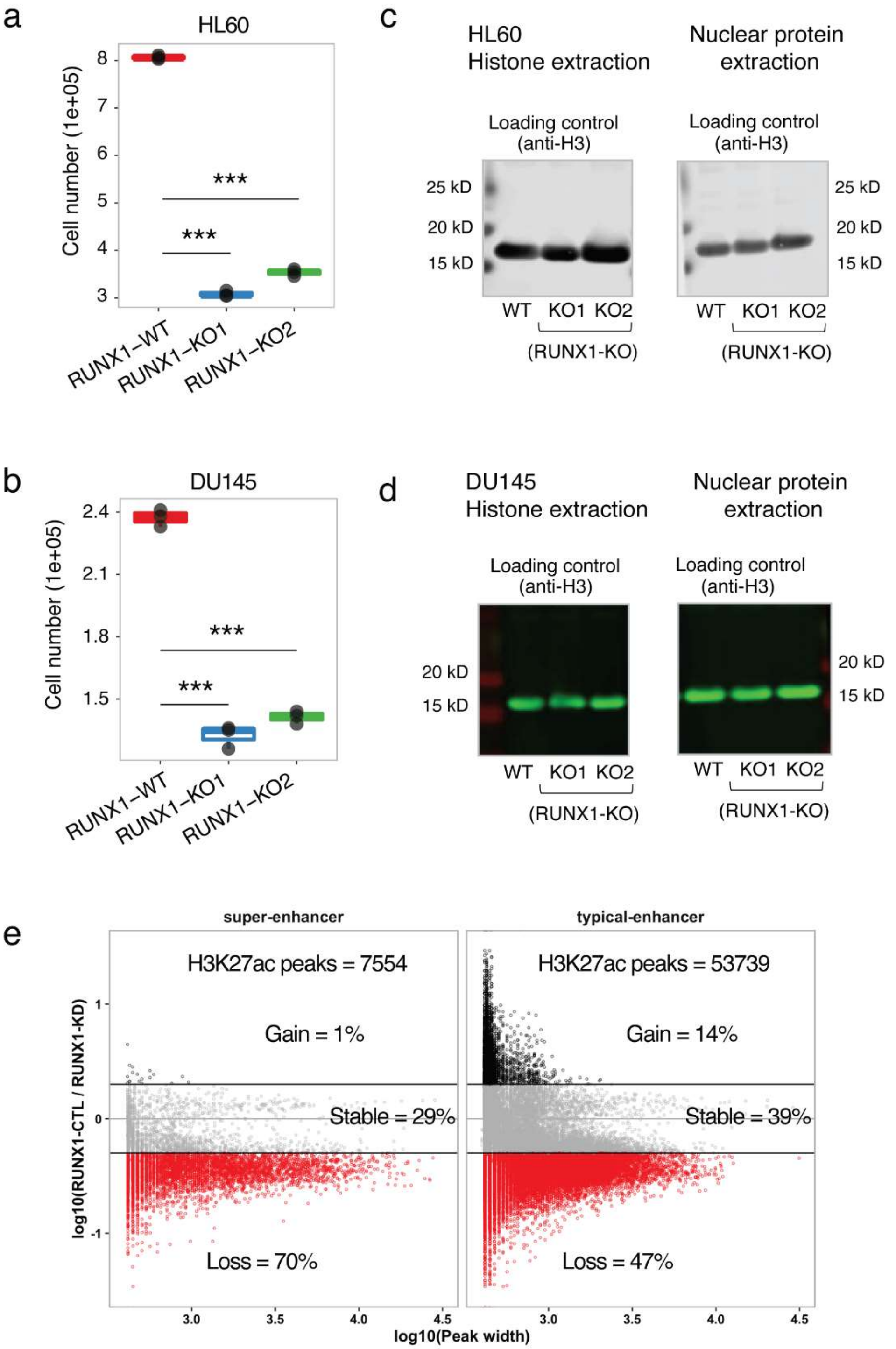
a
HL60
Cell number (1e+05)
8
7
6
5
4
3
***
***
RUNX1–WT
RUNX1–KO1
RUNX1–KO2
b
DU145
Cell number (1e+05)
2.4
2.1
1.8
1.5
***
***
RUNX1–WT
RUNX1–KO1
RUNX1–KO2
c
HL60
Histone extraction
Nuclear protein extraction
Loading control (anti-H3)
Loading control (anti-H3)
25 kD
20 kD
15 kD
25 kD
20 kD
15 kD
WT KO1 KO2
(RUNX1-KO)
WT KO1 KO2
(RUNX1-KO)
d
DU145
Histone extraction
Nuclear protein extraction
Loading control (anti-H3)
Loading control (anti-H3)
20 kD
15 kD
20 kD
15 kD
WT KO1 KO2
(RUNX1-KO)
WT KO1 KO2
(RUNX1-KO)
e
super-enhancer
typical-enhancer
H3K27ac peaks = 7554
Gain = 1%
Stable = 29%
Loss = 70%
H3K27ac peaks = 53739
Gain = 14%
Stable = 39%
Loss = 47%
log10(RUNX1-CTL / RUNX1-KD)
1
0
-1
3.0
3.5
4.0
4.5
log10(Peak width)

Figure S3: RUNX1 regulates global H3K27ac levels and super-enhancers. Related to Figures 1 and 2.

a,b) Number of live cells in control (RUNX1-WT) and RUNX1-KO samples at 72 hours in HL60 and DU145 cell lines. Cell numbers from three independent experiments are shown in boxplot. *P*-values were calculated using an unpaired two-tailed t-test. *** is the t-test p-value <= 0.05. **c,d)** Western blots for H3 loading control upon RUNX1-KOs in HL60 and DU145. **e)** In KOPTK1, normalized read densities of H3K27ac peaks are plotted in the scatter plots and peaks are divided based on their overlap with super-enhancers. H3K27ac peaks located outside of super-enhancers are considered as typical-enhancers. Each point represents a H3K27ac peak where the red point indicates >= 2-fold loss, black indicates >=2-fold gain and grey indicates stable peaks following RUNX1-KD. Read densities were normalized by sequencing depth.

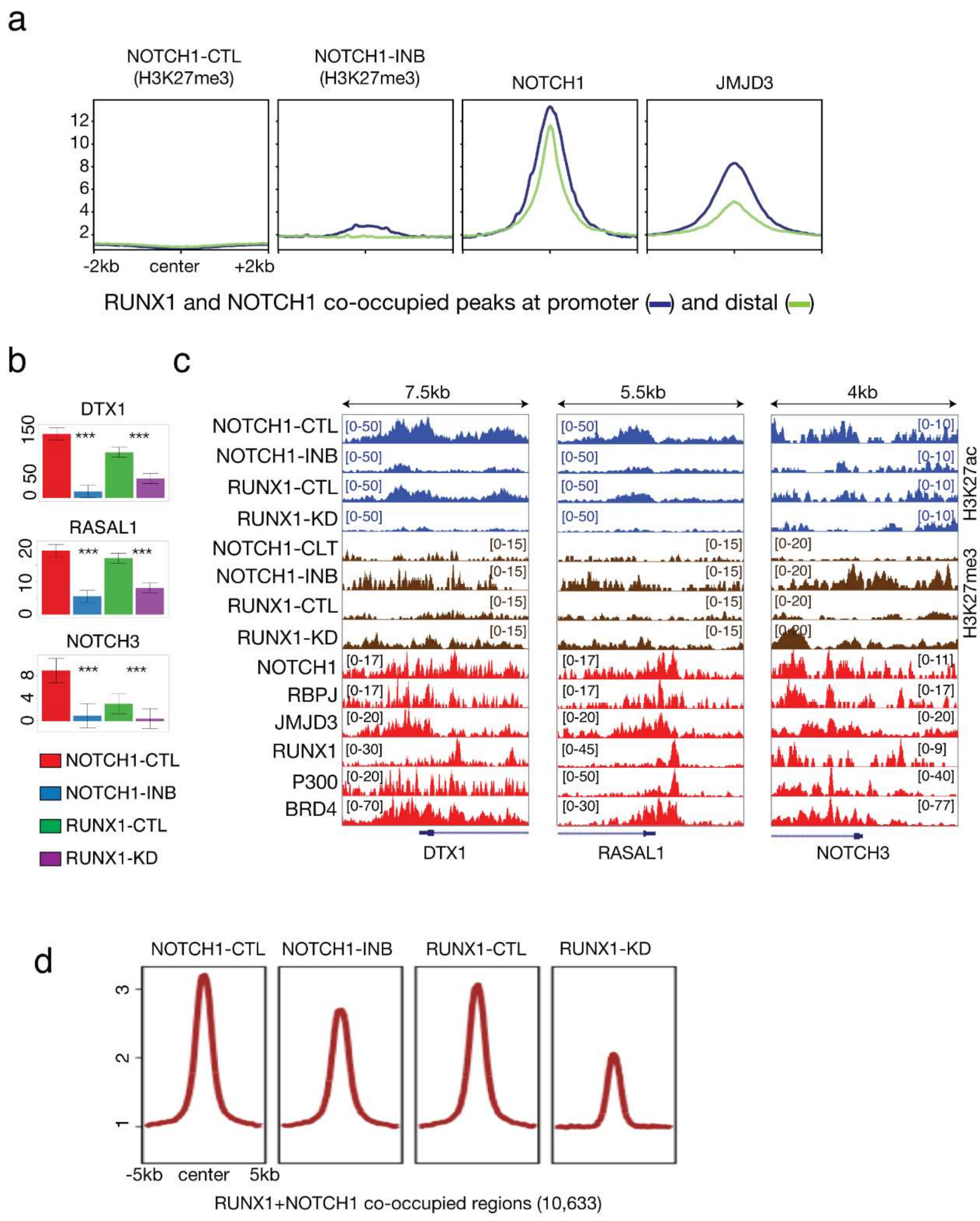
a
NOTCH1-CTL (H3K27me3)
NOTCH1-INB (H3K27me3)
NOTCH1
JMJD3
-2kb center +2kb
RUNX1 and NOTCH1 co-occupied peaks at promoter ( ) and distal ( )
b
DTX1
RASAL1
NOTCH3
NOTCH1-CTL
NOTCH1-INB
RUNX1-CTL
RUNX1-KD
c
7.5kb
5.5kb
4kb
NOTCH1-CTL
NOTCH1-INB
RUNX1-CTL
RUNX1-KD
H3K27ac
NOTCH1-CLT
NOTCH1-INB
RUNX1-CTL
RUNX1-KD
H3K27me3
NOTCH1
RBPJ
JMJD3
RUNX1
P300
BRD4
DTX1
RASAL1
NOTCH3
d
NOTCH1-CTL
NOTCH1-INB
RUNX1-CTL
RUNX1-KD
-5kb center 5kb
RUNX1+NOTCH1 co-occupied regions (10,633)

Figure S4: Cooperation between RUNX1 and NOTCH1. Related to Figures 3 and 4.

a) Mean coverage profiles for H3K27me3, NOTCH1 and JMJD3 at the RUNX1 and NOTCH1 co-occupied regions. **b)** The expressions of *DTX1, RASAL1* and *NOTCH3* are shown in two replicates of NOTCH1-INB and three replicates of RUNX1-KD in the KOPTK1 cell line. Data are mean ± s.e.m. of RPKM values and *** represents FDR <= 0.05 in differential gene expression analysis following RUNX1-KD or NOTCH1-INB. **c)** Normalized tracks for H3K27ac, H3K27me3, RUNX1, NOTCH1, RBPJ, JMJD3, P300 and BRD4 at the *DTX1, RASAL1* and *NOTCH3* regulatory loci. **d)** Mean coverage for H3K27ac upon RUNX1-KD and NOTCH1-INB around RUNX1+NOTCH1 co-occupied peaks. Signal intensity at the 5'end of the region was scaled to 1.

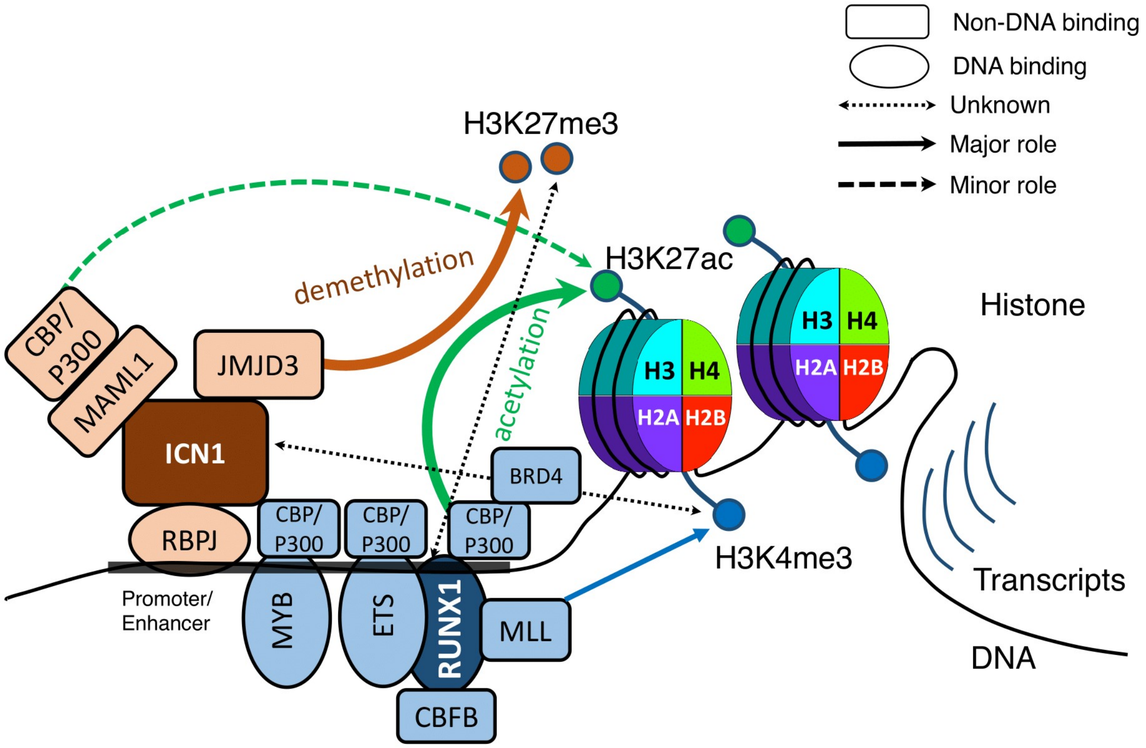

Figure S5: Cooperative activation of target genes by NOTCH1 and RUNX1. Related to Figures 3, 4 and S4.

A model of intracellular NOTCH1 (ICN1) and RUNX1 cooperativity. ICN1-JMJD3 plays major role in demethylation of H3K27me3 and RUNX1-P300 plays major role in depositing H3K27ac at the NOTCH1 and RUNX1 co-occupied regions to activate the transcription of their targets. Black dotted line indicates an unknown relationship.

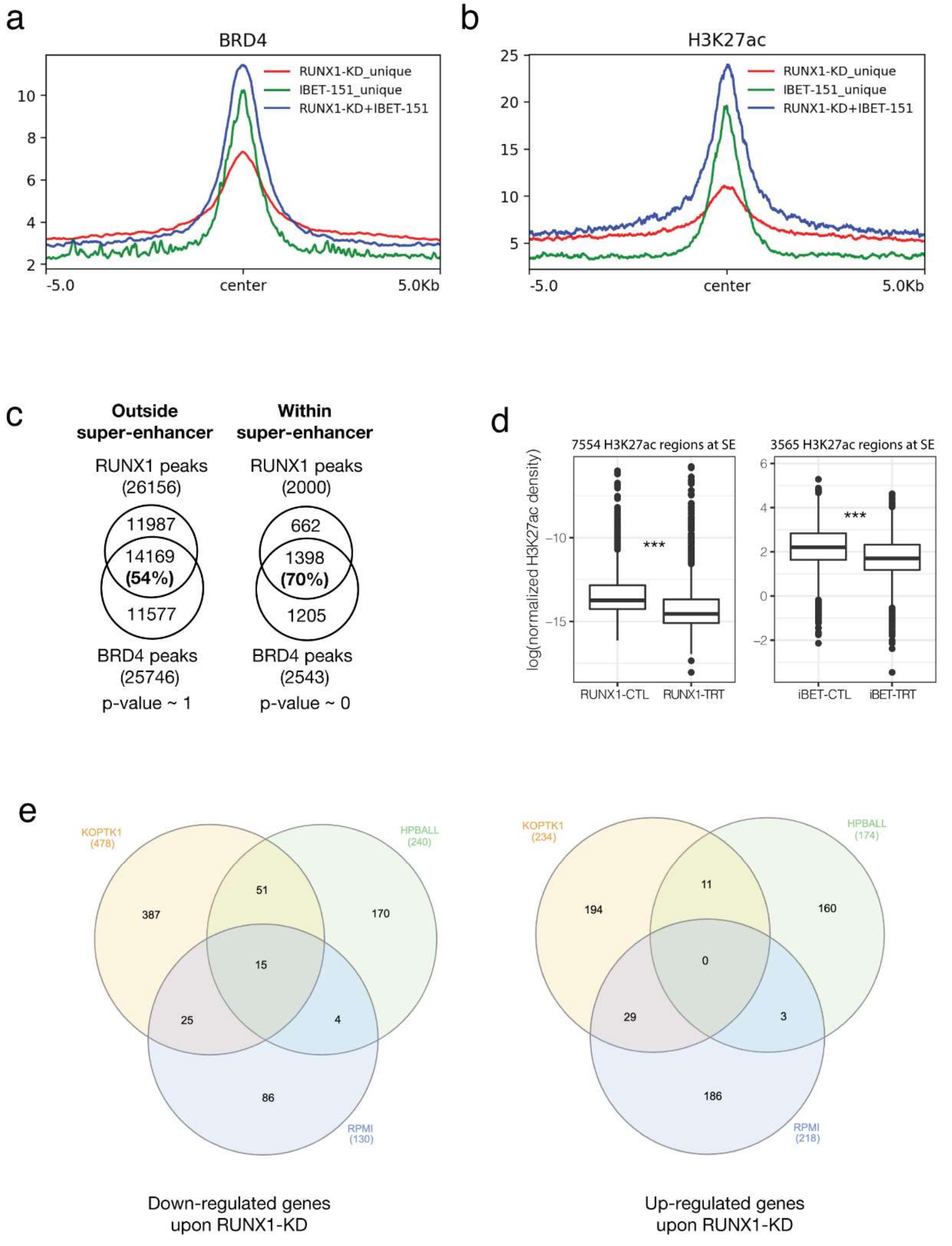
a
BRD4
RUNX1-KD_unique
IBET-151_unique
RUNX1-KD+IBET-151
-5.0
center
5.0Kb
b
H3K27ac
RUNX1-KD_unique
IBET-151_unique
RUNX1-KD+IBET-151
-5.0
center
5.0Kb
c
Outside super-enhancer
Within super-enhancer
RUNX1 peaks (26156)
11987
14169 (54%)
11577
BRD4 peaks (25746)
p-value ~ 1
RUNX1 peaks (2000)
662
1398 (70%)
1205
BRD4 peaks (2543)
p-value ~ 0
d
log(normalized H3K27ac density)
7554 H3K27ac regions at SE
***
RUNX1-CTL
RUNX1-TRT
3565 H3K27ac regions at SE
***
iBET-CTL
iBET-TRT
e
KOPTK1 (478)
HPBALL (240)
RPMI (130)
387
51
170
15
25
4
86
KOPTK1 (234)
HPBALL (174)
RPMI (218)
194
11
160
0
29
3
186
Down-regulated genes upon RUNX1-KD
Up-regulated genes upon RUNX1-KD

Figure S6: Cooperation between RUNX1 and BRD4. Related to Figures 5.

a,b) BRD4 and H3K27ac enrichment at the H3K27ac peaks that are sensitive to RUNX1-KD and/or I-BET151 treatment. RUNX1-KD+I-BET151 means the overlapping peaks that are lost by RUNX1-KD and I-BET151 treatment (n=8044). RUNX1-KD_unique (n=22674) or I-BET151_unique (n=5107) means the non-overlapping peaks that are uniquely lost by either RUNX1-KD or I-BET151 treatment. BRD4 ChIP-seq data were obtained from CUTLL1 T-ALL cell line and H3K27ac library (iBET-CTL) was generated in KOPTK1. **c)** Overlap of RUNX1 and BRD4 peaks at the super-enhancers identified in KOPTK1 cells. Statistical significance was estimated using Fisher's exact test. **d)** Normalized H3K27ac density within super-enhancers following RUNX1-KD or I-BET151 treatment in KOPTK1 cells. An unpaired two-tailed t-test was used to calculate the *p*-value. **e)** Overlap among the down-regulated and up-regulated genes following RUNX1-KD in three cell lines.

Table S1: Experimental strategies. Related to Figure 1.

NOTCH1-INB and RUNX1-KD experiments (RNA-seq, ChIP-seq for six histone marks e.g., H3K4me1, H3K4me3, H3K9me3, H3K27me3, H3K36me3, H3K27ac and input DNA control). Number of replicates are shown in brackets.

**Cell line (Synonyms)**	**Sample (# replicate)**	**Length of treatment**	**Assay**
KOPTK1 (KOPT-K1)	NOTCH1-CTL	3 days	RNA-seq, ChIP-seq (6 histone marks, input)
	NOTCH1-INB	3 days	RNA-seq, ChIP-seq (6 histone marks, input)
KOPTK1 (KOPT-K1)	RUNX1-CTL (non-silencing control)	5 days	RNA-seq, ChIP-seq (6 histone marks, input)
	RUNX1-KD (shRUNX1-58)	5 days	RNA-seq, ChIP-seq (6 histone marks, input)
	shRUNX1-59	5 days	RNA-seq, ChIP-seq (6 histone marks, input)
KOPTK1 (KOPT-K1)	RUNX1-CTL (2)	7 days	RNA-seq
	shRUNX1-58 (2)	7 days	RNA-seq
HPBALL (HPB-ALL)	RUNX1-CTL (2)	7 days	RNA-seq
	shRUNX1-58 (2)	7 days	RNA-seq
RPMI (RPMI 8402)	RUNX1-CTL (2)	7 days	RNA-seq
	shRUNX1-58 (2)	7 days	RNA-seq

Table S2: List of RUNX1 and NOTCH1 co-regulated genes. Related to Figure 3.

ABHD14A-ACY1	AEN	ARSG	ASB13	CBS	CD1A
CD300A	CISD3	COL6A3	CR2	CST7	DCAF4
DHCR7	DTX1	E2F5	EGR1	EIF4A1	EPHX2
FAM162A	FAM27A	FGR	FLJ00273	GLDC	GPR157
GPR17	HES4	IGF1R	IL4	IRF8	LPAR6
MFSD3	MMAB	MYC	MYO1B	MYO7B	NARS2
NAT10	NDST3	NOTCH3	PALD1	PDIA5	PELO
PEX5L	PKM	PMEPA1	POLR3G	PSAT1	PUS7
PYCR1	QDPR	RASAL1	RCBTB2	SCN7A	SEH1L
SLC38A5	SOWAHA	SQLE	SUSD4	TAF4B	TMA16
TRHDE	TXK	VARS	VCAN	WASF1	WDR3
XPO5	YBX3	KM-PA-2	ZNF593	AC004076.7	CCDC86
CD244	FAM86A	LZTFL1	NDUFAF2	NR1D1	TRUB2

Table S3: List of RUNX1, NOTCH1 and I-BET151 dependent genes. Related to Figure 5.

CD1A	FGR	IL4	NDST3	SCN7A	FAM162A
CD244	GLDC	IRF8	POLR3G	ASB13	PALD1
CD300A	GPR157	MYO7B	PYCR1	CST7	SLC38A5

Table S4: List of public datasets used in this study. Related to Figures 1-6.

Study/Sample	Cell line (tissue)	Assay	Reference
GSE51800	CUTLL1 (T-ALL)	ChIP-seq (NOTCH1, RBPJ, JMJD3, RUNX1, P300, BRD4)	[S1]
GSE78785	Primary T-ALL	RNA-seq	[S2]
ROADMAP	T-Cells	RNA-seq	[S3]
CEMT	HL60 (AML)	H3K27ac	https://thisisepigenetics.ca/data/CEMT/epi2021/grid/
CEMT	CLL	RNA-seq	[S4]
GSE68978	Jurkat (TALL)	ChIP-seq (H3K27ac, RUNX1, CBP)	[S5]
GSM1816978	ALL-SIL (T-ALL)	ChIP-seq (H3K27ac)	[S6]
GSM1519644	MOLT3 (T-ALL)	ChIP-seq (H3K27ac)	[S7]

Supplemental reference:

1. Wang, H., Zang, C., Taing, L., Arnett, K.L., Wong, Y.J., Pear, W.S., Blacklow, S.C., Liu, X.S., and Aster, J.C. (2014). NOTCH1-RBPJ complexes drive target gene expression through dynamic interactions with superenhancers. Proc. Natl. Acad. Sci. U. S. A. *111*, 705–710.
2. Andersson, A.K., Ma, J., Wang, J., Chen, X., Gedman, A.L., Dang, J., Nakitandwe, J., Holmfeldt, L., Parker, M., Easton, J., et al. (2015). The landscape of somatic mutations in infant MLL-rearranged acute lymphoblastic leukemias. Nat. Genet. *47*, 330–337.
3. Kundaje, A., Meuleman, W., Ernst, J., Bilenky, M., Yen, A., Heravi-Moussavi, A., Kheradpour, P., Zhang, Z., Wang, J., Ziller, M.J., et al. (2015). Integrative analysis of 111 reference human epigenomes. Nature *518*, 317–330.
4. Islam, R., Bilenky, M., Weng, A.P., Connors, J.M., Hirst, M., CRIS: complete reconstruction of immunoglobulin V-D-J sequences from RNA-seq data, Bioinformatics Advances, Volume 1, Issue 1, 2021, vbab021.
5. Hnisz, D., Weintraub, A.S., Day, D.S., Valton, A.-L., Bak, R.O., Li, C.H., Goldmann, J., Lajoie, B.R., Fan, Z.P., Sigova, A.A., et al. (2016). Activation of proto-oncogenes by disruption of chromosome neighborhoods. Science (80-.). *351*, 1454–1458.
6. Durinck, K., Van Loocke, W., Van der Meulen, J., Van de Walle, I., Ongenaert, M., Rondou, P., Wallaert, A., de Bock, C.E., Van Roy, N., Poppe, B., et al. (2015). Characterization of the genome-wide TLX1 binding profile in T-cell acute lymphoblastic leukemia. Leukemia *29*, 2317–2327.
7. Mansour, M.R., Abraham, B.J., Anders, L., Berezovskaya, A., Gutierrez, A., Durbin,

A.D., Etchin, J., Lawton, L., Sallan, S.E., Silverman, L.B., et al. (2014). An oncogenic super-enhancer formed through somatic mutation of a noncoding intergenic element. Science (80-.). *346*.
